# Elucidating the impact of microbial community biodiversity on pharmaceutical biotransformation during wastewater treatment

**DOI:** 10.1111/1751-7915.12870

**Published:** 2017-10-27

**Authors:** Lauren B. Stadler, Jeseth Delgado Vela, Sunit Jain, Gregory J. Dick, Nancy G. Love

**Affiliations:** ^1^ Department of Civil and Environmental Engineering University of Michigan Ann Arbor MI USA; ^2^ Department of Earth and Environmental Sciences University of Michigan Ann Arbor MI USA; ^3^ Present address: Department of Civil and Environmental Engineering Rice University 6100 Main Street, MS‐516 Houston TX 77005 USA; ^4^ Present address: Second Genome 341 Allerton Avenue South San Francisco CA 94080 USA

## Abstract

In addition to removing organics and other nutrients, the microorganisms in wastewater treatment plants (WWTPs) biotransform many pharmaceuticals present in wastewater. The objective of this study was to examine the relationship between pharmaceutical biotransformation and biodiversity in WWTP bioreactor microbial communities and identify taxa and functional genes that were strongly associated with biotransformation. Dilution‐to‐extinction of an activated sludge microbial community was performed to establish cultures with a gradient of microbial biodiversity. Batch experiments were performed using the dilution cultures to determine biotransformation extents of several environmentally relevant pharmaceuticals. With this approach, because the communities were all established from the same original community, and using sequencing of the 16S rRNA and metatranscriptome, we identified candidate taxa and genes whose activity and transcript abundances associated with the extent of individual pharmaceutical biotransformation and were lost across the biodiversity gradient. Metabolic genes such as dehydrogenases, amidases and monooxygenases were significantly associated with pharmaceutical biotransformation, and five genera were identified whose activity significantly associated with pharmaceutical biotransformation. Understanding how biotransformation relates to biodiversity will inform the design of biological WWTPs for enhanced removal of chemicals that negatively impact environmental health.

## Introduction

Wastewater treatment plants (WWTPs) harness microbes to treat human waste and protect our environment from organic pollutants, nutrients and pathogens. In addition to conventional pollutants, however, thousands of pharmaceuticals are excreted by humans in intact and metabolized forms, reaching WWTPs before being released into the environment (Kolpin *et al*., 2002). The ability of the microbes in WWTPs to biotransform these chemicals is an area of great interest (Carballa *et al*., 2004; Castiglioni *et al*., 2005; Nakada *et al*., 2006; Kasprzyk‐Hordern *et al*., 2009). Substantial research has advanced our knowledge of pharmaceutical biotransformation pathways (Ellis *et al*., 2006) and the transformation products formed during treatment (Kern *et al*., 2010). However, only one study to our knowledge has linked chemical transformation data with wastewater microbial community composition and activity (Helbling *et al*., 2015), and no studies to date have identified specific functions associated with biotransformation to develop predictive relationships between functional characteristics of the microbial community and pharmaceutical biotransformation pathways and extents.

Biodiversity is one characteristic of wastewater treatment microbial communities that may impact pharmaceutical biotransformation rates (Johnson *et al*., 2015). WWTPs harbour extremely diverse microbial communities (Zhang *et al*., 2011). Mounting evidence from studies of microbial systems suggests a positive relationship between biodiversity and the rates and/or magnitude of community functions (reviewed by Cardinale *et al*., 2006; Duffy, 2008). Functional redundancy, the concept that taxonomically distinct species have the same ecological function, challenges the idea that changes in biodiversity will directly affect community process rates. This is because processes that are carried out by many taxonomically distinct microorganisms, or broad processes, would not necessarily be impacted by biodiversity losses as the process rate is not limited by the number of species that can perform it. Conversely, narrow processes, or processes performed by few species, would be positively correlated with biodiversity because the process rate is limited by the number of species able to perform the specialized metabolism.

In wastewater systems, biodegradation processes may be broad or narrow depending on the compound under consideration. Given the diverse chemical structures of pharmaceuticals, their biotransformation could be catalysed by either broad or narrow processes. In a study of microbial communities from ten full‐scale treatment systems, a positive association between taxonomic and functional biodiversity and the rates of some compounds was observed (Johnson *et al*., 2015). However, not all compound biotransformation rates in this study exhibited a positive association with biodiversity; those compounds were likely transformed by broad processes. In laboratory‐manipulated bioreactors, Pholchan *et al*. (2013) found that communities with greater diversity were associated with a decrease in the removal of a suite of estrogens [17‐estradiol (E2), estrone (E1), estriol (E3) and 17α‐ethinylestradiol (EE2)], which was counterintuitive to their hypothesis. They concluded that it was not possible to make blanket statements about the relationship between rare functions and biodiversity. Conversely, Hernandez‐Raquet *et al*. (2013) found that dilution‐induced reduction in diversity of an activated sludge community resulted in significant reductions in phenanthrene mineralization. These studies collectively show that while positive biodiversity–function relationships may hold for specific pharmaceutical biotransformations and collective pharmaceutical biotransformation, more resolved information, such as the relative activity of specific taxa and/or functions, is needed to understand conflicting patterns observed for individual pharmaceutical compounds. Understanding whether pharmaceutical biotransformations are catalysed by highly redundant populations or performed by rare taxa can help us identify the enzymes that catalyse their transformation and exploit opportunities for enhancing biotransformations during wastewater treatment.

The goal of this study was to elucidate a more resolved understanding of why and how biodiversity affects pharmaceutical biotransformation by experimentally manipulating the biodiversity of wastewater microbial communities. We manipulated an activated sludge community using a dilution‐to‐extinction approach to create communities with different levels of biodiversity and directly test the relationship between biodiversity and function (here, defined as pharmaceutical biotransformation). With this approach, because the communities were all established from the same original community, we hypothesized that we could identify taxa and differentially expressed genes that were correlated with pharmaceutical biotransformation. These taxa and genes can serve as predictive biomarkers for pharmaceutical biotransformation, and WWTPs could be designed or operated to enhance the activity of these taxa and functions to improve overall pharmaceutical biotransformation by wastewater microbial communities.

## Results

### Dilution resulted in communities with different levels of biodiversity

A gradient in microbial biodiversity in an activated sludge community was established using a dilution‐to‐extinction approach (Szabó *et al*., 2007; Peter *et al*., 2010; Philippot *et al*., 2013; Ylla *et al*., 2013). In this approach, each dilution theoretically removes the least abundant species from the previous culture, resulting in a less diverse subset of the original community. Activated sludge was serially diluted stepwise (1:10) in sterile semi‐synthetic sewage media (SSM) to achieve dilution conditions from 10^−1^ to 10^−7^. After serial dilution, triplicate flasks of the 10^−2^, 10^−4^ and 10^−7^ dilutions were allowed to regrow overnight such that all the dilution cultures were a similar abundance. After regrowth, the biomass was pelleted and re‐suspended in fresh SSM before performing the pharmaceutical biotransformation batch experiments. We quantified the loss of seven pharmaceuticals and normalized the loss of each compound by the biomass concentration and time elapsed between initial and final sample collection for each batch. Biomass samples were collected from each batch for DNA and RNA extractions and sequencing. The 16S rRNA gene and 16S rRNA sequencing data were used to calculate the DNA‐ and RNA‐based taxonomic biodiversity measurements, respectively, and the shotgun metagenomic and metatranscriptomic sequencing data were used to generate the DNA‐ and RNA‐based functional biodiversity measurements respectively.

**Figure 1:**
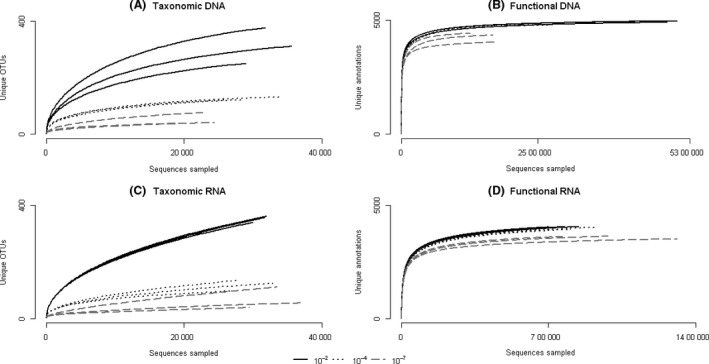
Rarefaction plots for the dilution cultures based on 16S rRNA gene and 16S rRNA sequencing (taxonomic) and metagenomic and metatranscriptomic sequencing (functional). Dilution conditions are shown in black (10^−2^), dark grey, dotted (10^−4^) and light grey, dashed (10^−7^).

With increased dilution, there was a decrease in the taxonomic richness of the activated sludge microbial community based on both 16S rRNA gene and 16S rRNA sequencing (ANOVA, *P *<* *0.05). Rarefaction curves of unique operational taxonomic units (OTUs; grouped based on sequence similarity of greater or equal to 97%) based on both DNA and RNA sequencing showed distinct clustering of the dilution cultures, with samples from the most diverse culture (10^−2^) having the greatest number of unique OTUs, followed by the medium‐biodiversity culture (10^−4^) and the low‐biodiversity culture (10^−7^) that plateaued with the lowest number of unique OTUs (Fig. 1A and C). Differences in DNA‐based taxonomic diversity between the dilution cultures are supported by various biodiversity indices based on the 16S rRNA gene sequence data (Table 1).

**Table 1:** Biodiversity indices based on 16S rRNA gene, 16S rRNA, metagenomic and metatranscriptomic sequencing of biomass from the dilution cultures. The same letter indicates treatments without significant differences based on pairwise comparisons (*t*‐test, Bonferroni‐adjusted two‐sided *P *>* *0.05). Reported values are averages and standard deviations of triplicate batches

Biodiversity index	Dilution condition		
	10^−2^	10^−4^	10^−7^
DNA			
Taxonomic richness (unique OTUs)	311 ± 63.0^A^	123 ± 6.66^B^	51.0 ± 20.8^C^
Chao1 extrapolated taxonomic richness	358 ± 67.8^A^	136 ± 3.75^B^	64.1 ± 23.4^C^
Shannon taxonomic diversity	2.67 ± 0.189^A^	2.02 ± 0.0619^B^	1.37 ± 0.0594^C^
Pielou taxonomic evenness	1.05 ± 0.0547^A^	0.944 ± 0.0239^A,B^	0.773 ± 0.0902^B^
Functional richness (unique functional genes)	4600 ± 45.0^A^	4560 ± 27.0^A^	4130 ± 221^A^
Chao1 extrapolated functional richness	4760 ± 12.9^A^	4720 ± 46.9^A^	4240 ± 181^B^
Shannon functional diversity	7.64 ± 0.0103^A^	7.60 ± 0.00258^B^	7.55 ± 0.00905^C^
Pielou functional evenness	2.08 ± 0.00332^A^	2.07 ± 0.00308^A^	2.08 ± 0.00823^A^
RNA			
Taxonomic richness (unique OTUs)	512 ± 9.54^A^	190 ± 21.7^B^	109 ± 35.9^B^
Chao1 extrapolated taxonomic richness	983 ± 61.9^A^	354 ± 31.7^B^	208 ± 84.4^B^
Shannon taxonomic diversity	2.71 ± 0.0407^A^	1.95 ± 0.0406^B^	1.62 ± 0.122^C^
Pielou taxonomic evenness	0.906 ± 0.0211^A^	0.767 ± 0.00654^B^	0.710 ± 0.102^A,B^
Functional richness (unique functional genes)	3930 ± 20.3^A^	3820 ± 20.1^B^	3420 ± 106^C^
Chao1 extrapolated functional richness	4220 ± 24.1^A^	4140 ± 53.6^A^	3650 ± 115^B^
Shannon functional diversity	6.51 ± 0.0590^A^	6.56 ± 0.120^A^	6.53 ± 0.0369^A^
Pielou functional evenness	1.80 ± 0.0175^A^	1.82 ± 0.0310^A^	1.83 ± 0.00627^A^

We did not observe significant differences in DNA‐based functional richness between the dilution conditions (Fig. 1B, Table 1; *t*‐test, Bonferroni‐adjusted two‐sided *P *>* *0.05), in contrast to the DNA‐based taxonomic richness measurements. Conversely, RNA‐based functional richness (Fig. 1D) was significantly different between each dilution condition based on pairwise comparisons (*t*‐test, Bonferroni‐adjusted two‐sided *P *<* *0.05). Overall, differences in biodiversity measurements were not consistent between DNA‐ and RNA‐based approaches, or between taxonomic and functional datasets.

### Taxonomic and functional diversity positively associated with one another

To understand if more unique taxa corresponded to increased functional traits in the wastewater microbial communities, we first tested whether taxonomic richness was positively associated with functional richness. We found that for both the DNA‐ and RNA‐based annotations, there was a significant positive association between taxonomic and functional richness (Fig. S2; Spearman, *P *<* *0.005). The shape of the data in Fig. S2 suggests that the number of unique functions does not increase linearly with the number of unique OTUs and instead the number of functions levels out at high numbers of OTUs. This is consistent with the idea that the most diverse communities are also the most functionally redundant and that the unique OTUs contain many of the same functional genes.

### Carbon oxidation and pharmaceutical transformations show different patterns with dilution

Carbon utilization, a process that is widespread across all forms of life, is a function that we expected to be functionally redundant across all the dilution conditions. Thus, we hypothesized that there would not be a significant difference in specific carbon oxidation rates between the dilution conditions. To test this, dissolved organic carbon was measured in samples across the experiment to determine carbon oxidation rates (Fig. S3). Indeed, we found no significant difference between volatile suspended solids (VSS) – normalized carbon oxidation rates across all dilution conditions (ANOVA, *P *>* *0.64). In all dilution conditions, we found no significant association between specific carbon oxidation rate and taxonomic or functional richness (Spearman, *P *>* *0.80), supporting the notion that carbon oxidation is a function that is widespread and redundant in wastewater microbial communities (Franklin and Mills, 2006).

**Figure 2:**
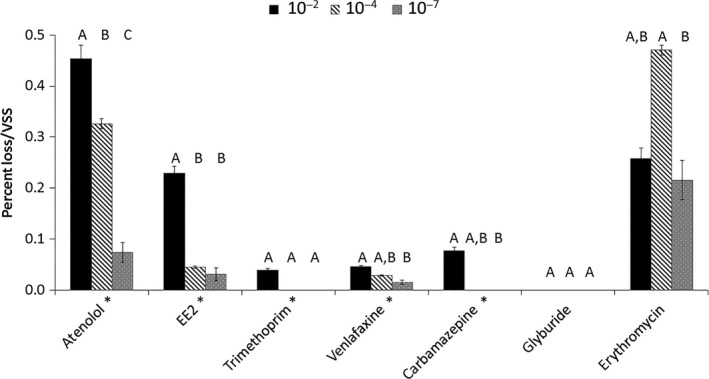
Average pharmaceutical loss (disappearance of the parent compound, *n* = 3) normalized to volatile suspended solids concentration for each dilution condition (black: 10^−2^; dark grey hatch: 10^−4^; light grey: 10^−7^). The asterisk (*) by the compound name indicates a significant difference among the group means (ANOVA,* P *<* *0.05). The same letters above the bars indicate treatments without significant differences between biotransformation extent (*t‐test,* Bonferroni‐adjusted two‐sided *P *>* *0.05). Error bars represent standard deviations of triplicate batches.

In contrast to specific carbon oxidation rate patterns, significant differences between the degree of biotransformation and the dilution condition were observed for five of the seven pharmaceutical compounds, with greater extents of biotransformation observed in the most diverse culture (ANOVA, *P *<* *0.05; Fig. 2). Two compounds did not follow the same pattern as the rest: for glyburide, no significant differences in extent of biotransformation between the dilution cultures were observed as very limited loss of the parent compound occurred; and for erythromycin, the greatest loss was observed in the 10^−4^ condition, although it was not statistically significantly different from the 10^−2^ condition (*t‐*test, Bonferroni‐adjusted *P *>* *0.05). Atenolol was the only compound for which the stepwise dilution resulted in a corresponding stepwise reduction in biotransformation. For the other compounds, with the exception of trimethoprim (for which there was no significant differences between conditions based on pairwise t‐tests), there was only a significant difference in biotransformation extent between the 10^−2^ and the other dilution conditions.

### Significant associations between functional richness and pharmaceutical biotransformation extent were observed

**Figure 3:**
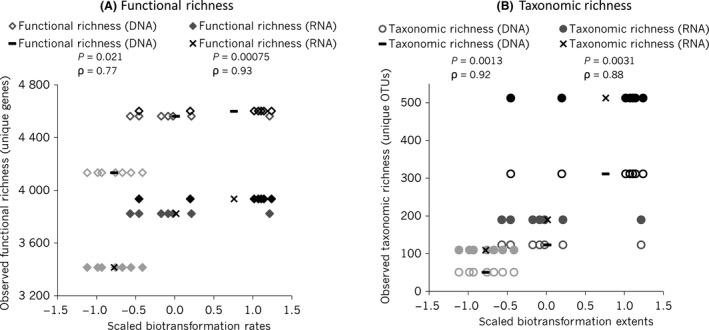
Relationship between richness and pharmaceutical biotransformation extent. Left (A) represents the functional richness, and right (B) represents taxonomic richness. Each diamond or circle represents a different pharmaceutical compound. Open diamonds and circles represent DNA‐based richness and filled diamonds, and circles represent RNA‐based richness. The 10^−7^ dilution condition (least diverse) is represented in light grey, the 10^−4^ condition is in grey, and the 10^−2^ condition (most diverse) is in black. The average transformation extents across all compounds are shown with a black line (DNA‐based richness) and a cross (RNA‐based richness). Reported *P*‐values and ρ (rho) values (Spearman rank correlation coefficients) are based on a two‐sided Spearman rank correlation test.

We observed a significant positive association between DNA‐ and RNA‐based functional richness measurements and scaled pharmaceutical biotransformation extents (Spearman, *P *<* *0.05; Fig. 3). Notably, RNA‐based functional richness was a better predictor of pharmaceutical biotransformation extent than DNA‐based functional richness based on Spearman rank correlation coefficient values (ρ_RNA‐func‐richness_ = 0.93, ρ_DNA‐func‐richness_ = 0.77). This indicates that expressed genes are better predictors of pharmaceutical biotransformation and supports the notion that metagenomic datasets may mask significant associations with magnitudes of community functions as they include non‐expressed traits. The observed functional richness appears to plateau (Fig. 3A), particularly between the 10^−2^ and 10^−4^ conditions, whereas the observed taxonomic richness does not (Fig. 3B), again indicative of a large degree of functional redundancy in the most diverse microbial communities. We chose to focus on RNA‐based taxonomic and functional richness when assessing associations with pharmaceutical biotransformation and to identify lost active OTUs and expressed functional genes across the dilution conditions.

### Differential expression of functional genes suggests potential enzymes associated with biodegradation

After observing that there were significant positive associations between biodiversity and pharmaceutical biotransformation extent, we asked which functions were differentially expressed across the dilution cultures, and specifically which functions were lost? Of the 710 402 genes with predicted functions analysed from the combined assembly, 15 290 were found to be differentially expressed between the three dilution conditions (Benjamini–Hochberg adjusted *P *<* *0.05) and to have matches in the KEGG Orthology database. Differential expression could have been due to differences in transcript abundances, or to gene absence in the different dilution conditions. The majority of the significantly differentially expressed functions were lost with dilution from the 10^−2^ to the 10^−7^ cultures (negative log fold change, Fig. S6). For the compounds that were transformed to different extents across the three dilution conditions, the genes that had a lower level of expression in the least diverse culture (10^−7^) could be responsible for pharmaceutical biotransformation.

We sought to establish whether genes that might have been involved in pharmaceutical biotransformation were differentially expressed across the dilution conditions and thus focused on compounds that were transformed to a different extent with increased dilution (atenolol, 17α‐ethinylestradiol (EE2), trimethoprim, venlafaxine, erythromycin and carbamazepine). For all compounds except erythromycin, we further narrowed the list of genes relevant to those compounds by selecting genes only if they were lost with increased dilution. The list of genes was narrowed by focusing on classes of metabolic genes that were predicted to be involved in the compound's biotransformation by the EAWAG‐BBD Pathway Prediction System (Ellis *et al*., 2006; EAWAG, 2016). The classes of metabolic genes included were those with functional assignments for genes which transcribe the following enzymes: amidase, nitrilase, transaminase, demethylase, oxidase, hydrolase, dehydrogenase, aminotransferase, monooxygenase, hydroxylases, esterase, lactonase, amidohydrolase, dioxygenase, oxygenase, lactamase, sulfatase and dimethylamine hydrogenase. Spearman rank correlation tests between the normalized expression of each of the genes that were predicted to be involved in biotransformation and each compound's biotransformation extent were performed to identify genes with expression patterns that were significantly associated with biotransformation extent. The list of statistically significant genes for each compound is given in Table S6.

Gene set enrichment analysis was used to understand which KEGG functions were more likely to be positively or negatively associated with biotransformation across all the compounds analysed. By comparing entire categories of functions, rather than individual genes, the statistical power of the analysis increased. This analysis also allowed us to evaluate which KEGG functional group categories were biomarkers for overall transformation (Table S7), rather than just statistically correlated with transformation of each individual compound. Based on a literature review, there is previous evidence for use of many of the associated genes as biomarkers for aromatic compound degradation. A heat map showing expression of genes annotated with these KEGG categories across the different dilution conditions is shown in Fig. S6.

### Active taxa or operational taxonomic units (OTUs) that associated with biotransformation extent were identified

We identified specific OTUs that may have been involved in pharmaceutical biotransformation and were lost across the dilution conditions. Using the 16S rRNA sequencing results, we identified OTUs whose activity (abundance of 16S rRNA gene transcripts) significantly associated with individual and collective pharmaceutical biotransformation extents (Table 2). We narrowed the list of significantly associated OTUs to those that had greater than 0.5% average relative activity in the 10^−2^ condition. Five OTUs whose activity significantly associated with individual and/or collective pharmaceutical biotransformation extents were identified (Table 2). While this approach does not allow us to definitively conclude that these taxa are involved in biotransformation (e.g. they could simply co‐occur with taxa that perform the biotransformation reactions), they can be viewed as a list of useful biomarkers that are predictive of biotransformation. Based on a literature review of the associated OTUs (references provided in Table 2), all have previously been identified in biodegradation studies, either as directly involved in the biotransformation of a pollutant, or identified in systems performing aerobic pollutant degradation, thus supporting their potential importance in catalysing pharmaceutical biotransformations.

**Table 2:** Phylogenetic assignments of OTUs with relative activities that significantly associated with pharmaceutical biotransformation extents

Phylum	Class	Order	Family	Genus	Compound(s)	Literature supporting role in biotransformation
*Proteobacteria*	*Betaproteobacteria*	*Neisseriales*	*Neisseriaceae*	*Vogesella*	Atenolol, venlafaxine, collective	(Pérez‐Pantoja *et al*., 2010; Arroyo‐Caraballo and Colon‐Burgos, 2000)
*Bacteroidetes*	*Flavobacteriia*	*Flavobacteriales*	*Weeksellaceae*	*Cloacibacterium*	Atenolol, collective	(Amorim *et al*., 2013; Allen *et al*., 2006)
*Proteobacteria*	*Betaproteobacteria*	*Burkholderiales*	*Comamonadaceae*	*Acidovorax*	Atenolol, venlafaxine	(Martínková and Křen, 2010)
*Bacteroidetes*	*Flavobacteriia*	*Flavobacteriales*	*Weeksellaceae*	*Chryseobacterium*	Atenolol, venlafaxine	(Helbling *et al*., 2015; Jobanputra *et al*., 2011; Takenaka *et al*., 2013)
*Bacteroidetes*	*Flavobacteriia*	*Flavobacteriales*	*Flavobacteriaceae*	*Flavobacterium*	Atenolol, venlafaxine, collective	(Crawford and Mohn, 1985; Tweel *et al*., 1988)

## Discussion

Despite a wealth of both pharmaceutical loss data across WWTPs and activated sludge sequencing data, we lack robust datasets that allow us to link the two sets of information. Further studies are needed to generate candidate lists of taxa that might be used as predictive biomarkers for pharmaceutical biotransformation so that we may be able to design and operate WWTPs to enhance biotransformation and address emerging water quality goals. While a previous study looked at the relationship between biodiversity and pharmaceutical biotransformation in WWTPs, they did not go beyond testing associations with whole community biodiversity measurements. To gain a more mechanistic understanding of (i) why certain pharmaceuticals have strong positive associations with taxonomic and/or functional biodiversity; and (ii) how microbial community structure and activity influences pharmaceutical biotransformation, we need to look at which specific functions and taxa are strongly associated with pharmaceutical loss. In this study, we go beyond bulk biodiversity measurements and identified both functional genes (Table S7) and OTUs (Table 2) whose activity significantly associated with pharmaceutical biotransformation. As the communities were all established from the same original community and diversity manipulations were achieved with a dilution‐to‐extinction approach, we could examine how the loss of specific functions and taxonomic groups affected pharmaceutical biotransformation. Further, by comparing the relative activity of OTUs and the expression of functional genes across the dilution conditions, we identified OTUs and functional genes whose presence and expression correlated with biotransformation. The dataset generated in this study represents a resource for future studies that seek to link WWTP community structure and activity to pharmaceutical biotransformation.

### Taxonomic and functional richness associated with pharmaceutical biotransformation, and RNA‐based richness had stronger associations than DNA‐based richness measurements

We saw a strong positive association between both taxonomic and functional biodiversity and overall biotransformation extent (Fig. 3), similar to the findings of Johnson *et al*. (2015). These results are also consistent with microbial communities studied in WWTPs and other environments that showed that communities with more taxa are likely to have more functional traits (Gilbert *et al*., 2010; Bryant *et al*., 2012; Johnson *et al*., 2014). We also observed functional redundancy as the shape of the association between taxonomic and functional genes was not linear (Fig. S2), and thus, the most diverse communities likely had significant redundancy with respect to functional genes. We chose to use RNA‐based functional and taxonomic richness measurements for testing associations with pharmaceutical biotransformation as RNA excludes non‐expressed traits and captures the active fraction of the community. We observed differences in biodiversity measurements between DNA‐ and RNA‐based approaches; for example, RNA‐based functional richness was significantly different between the dilution conditions, but not significantly different based on DNA (Table 1). This indicated that our RNA‐based approach could potentially capture more pronounced differences in expressed functions between the dilution conditions.

We found that pharmaceutical biotransformation rates were significantly associated with both functional and taxonomic richness (Fig. 3). This indicates that for the purpose of understanding the relationship between biodiversity and function, amplicon sequencing of the 16S rRNA was a sufficient measure of biodiversity to test associations with process rates. This may not hold true in highly functionally redundant microbial communities, where expressed taxonomic and functional diversity are not strongly associated with one another (e.g. Ylla *et al*., 2013). Using 16S rRNA to test relationships with biodiversity is advantageous because amplicon sequencing is more affordable and less computationally intensive, generates a fraction of the data, and has more developed reference databases compared with functional genes. However, only by performing metagenomic and metatranscriptomic sequencing is it possible to test associations with specific genes and generate candidate gene lists that can be used to discover mechanistic links with biotransformation.

### The taxa and functional genes that associated with pharmaceutical biotransformation were consistent with previous research and form a basis for testing causal relationships

We identified five OTUs whose activity significantly associated with individual compound biotransformation extents (atenolol and/or venlafaxine, Table 2). All the OTUs have previously been identified in biodegradation processes. Helbling *et al*. (2015) also found a significant association between venlafaxine and the activity of *Chryseobacterium*, despite differences in experimental design (fed vs. starved batch conditions) and using different WWTP biomass. This supports the validity of our approach and suggests that the OTUs identified may serve as useful biomarkers across a range of wastewater environments. Further, taxonomic diversity may underpin faster, more resilient and more robust processes because the different microbial community members have different properties (e.g. substrate affinities, energy and nutrient requirements). While many different organisms may express similar genes and are capable of biotransforming pharmaceuticals, specific groups of organisms may be larger contributors to overall transformation rates. For example, Khunjar *et al*. (2011) found that both ammonia oxidizing bacteria (AOB) and heterotrophs were capable of catalysing the hydroxylation of EE2, likely using a monooxygenase enzyme, but AOB perform the process more rapidly than heterotrophs. Thus, understanding ‘who’ is performing the function may be more important than the expression of the relevant functional gene to understand what controls a biotransformation rate. This may also explain why we saw a strong association between atenolol biotransformation and functional richness. Based on our knowledge of atenolol biotransformation in aerobic systems (hydrolysis of the primary amide, Table S2), we might expect that it would be a relatively broad process. However, if the rate of the primary amide hydrolysis differs highly between taxa, then the positive association between atenolol biotransformation and taxonomic biodiversity would hold and in turn also be positively associated with functional richness because of the positive association between taxonomic and functional richness.

Beyond taxonomic data, we can use metagenomic and metatranscriptomic sequencing to test associations between pharmaceutical biotransformation extent and gene activity and generate candidate gene lists (Table S6) that can be used to discover mechanistic links with biotransformation. In this study, associated genes were extensive, and the number of associated KEGG orthologs ranged from 5 to 156, depending on the compound. Therefore, the data generated from these associations are intended to be hypothesis‐generating and elucidate targets for further study. To focus these targets, we used gene set enrichment analysis to identify KEGG functional groups that were statistically associated with pharmaceutical transformations across all of the compounds that had loss of transformation at increased dilution (Table S7 and Fig. S6). Twenty‐eight of the 39 KEGG functional groups that were significantly associated with pharmaceutical transformation encoded for dehydrogenase enzymes. In addition, many of the significantly associated genes encoded functions that are part of central metabolic pathways such as oxidative phosphorylation, amino acid metabolism and the TCA cycle. These associations were likely significant because these KEGG categories represented those that associated with transformation of all the compounds. Genes catalysing more specific initial transformations were associated with the transformation of individual compounds. For example, the gene encoding for subunit C of the ammonia monooxygenase gene was associated with EE2 biotransformation (Benjamini–Hochberg adjusted *P *=* *0.025, ρ = 0.69, adjusted for multiple comparisons), as we would expect given our knowledge of the transformation pathway (Khunjar *et al*., 2011). In addition, genes encoding amidase enzymes were associated with atenolol transformation (Helbling *et al*., 2010) (Benjamini–Hochberg adjusted *P *<* *0.03, ρ > 0.78). The consistency of these results with other previous studies provides some validity to our approach. After initial biotransformation reactions, pharmaceutical compounds are eventually broken down into central intermediates, which may explain the increased expression of genes that encode general metabolic pathway functions. This is consistent with previous studies that observed increased expression of genes in relation to amino acid metabolism, TCA cycle and oxidative phosphorylation in microbial processes degrading organic contaminants (Annweiler *et al*., 2000; Li *et al*., 2012).

### Enhancing biodiversity in wastewater treatment plants could enhance overall biotransformation of pharmaceuticals

For those compounds for which we saw no significant difference between loss and dilution condition (e.g. erythromycin), increased biodiversity is not likely a successful strategy for achieving enhanced removal. Interestingly, for most the compounds studied (5 of 7) their extent of loss decreased with increased dilution (Fig. 2). One way to enhance the loss of compounds that undergo these types of biotransformation processes may be to design WWTPs to support diverse microbial communities and harness specific low‐abundance community members. Recent studies suggest that certain WWTP operational parameters, such as solids retention time (Vuono *et al*., 2016), dissolved oxygen conditions (Stadler and Love, 2016), and concentration and composition of dissolved organic matter (Li *et al*., 2014) can all influence the degree of microbial biodiversity and the extent of micropollutant removal. Further research is needed to understand the relative importance of these operational parameters and environment conditions and others on WWTP microbial biodiversity and pharmaceutical biotransformation.

In conclusion, we observed significant positive associations between biodiversity of WWTP microbial communities and pharmaceutical biotransformation. By linking gene expression and relative activity with individual pharmaceutical biotransformation extents, this work goes beyond testing associations between biodiversity measurements and pharmaceutical biotransformation to identify metabolic genes and OTUs which can be potentially used as biomarkers for biotransformation. Metabolic genes such as dehydrogenases, amidases and monooxygenases were significantly associated with pharmaceutical biotransformation. Five genera were identified whose activity significantly associated with pharmaceutical biotransformation (Table 2), and previous studies support their potential involvement in catalysing biotransformation. Further experimentation is needed to conclusively link those functions and taxa to biotransformation reactions. The strong positive association between biodiversity and pharmaceutical biotransformation extent has implications for the design and operation of WWTPs to increase pharmaceutical removal. Specifically, creating niche environments that support the growth of diverse microbial communities could result in better overall performance with respect to pharmaceutical removal. Understanding the factors that drive biodiversity and enhance the activity of key populations involved in biotransformation is needed to harness the benefits of biodiversity for wastewater treatment.

## Experimental procedures

### Experimental design and biodiversity manipulation

An 8‐l grab sample of activated sludge mixed liquor was collected from the aeration basin of the Ann Arbor WWTP, a facility that performs nitrification and moderate biological phosphorus removal. Biodiversity manipulations were achieved using a dilution‐to‐extinction approach. Dilution‐induced reductions in diversity have been used in numerous previous studies to understand structure–function relationships in mixed microbial communities (e.g. Franklin and Mills, 2006; Hernandez‐Raquet *et al*., 2013; Philippot *et al*., 2013). Before serial dilution, disaggregation of macroflocs in the activated sludge sample was achieved by blending approximately 500 ml of mixed liquor in an industrial blender (Waring Commercial Blender, Model 516L31) at maximum speed for 10 min. After blending, stepwise dilutions (1:10) were performed by transferring 100 mL into 900 mL of sterile semi‐synthetic sewage media (SSM) to achieve dilution conditions from 10^−1^ to 10^−7^. The SSM was comprised of filtered (0.22 μm Stericup, Millipore, Darmstadt, Germany) and autoclaved primary effluent collected from the Ann Arbor WWTP (detailed in Appendix S1). Chemical oxygen demand (COD) was determined in the filtered and sterilized primary effluent using Standard Methods (2005) and then supplemented with carbon (a mixture of peptone, meat extract and humic acid) and ammonium chloride to achieve a final concentration of 1850 mg l^−1^ as COD and 30 mg‐N l^−1^ as ammonium. The COD concentration was selected to maintain ‘fed’ conditions throughout a significant portion of the batch experiment. After serial dilution, triplicate flasks of 200 mL of the 10^−2^, 10^−4^ and 10^−7^ dilutions were allowed to regrow (approximately 14 h) in an incubator‐shaker at 20°C. Regrowth was performed to allow each of the dilution cultures to reach a similar abundance, and biomass was pelleted and re‐suspended in fresh SSM prior adding the pharmaceutical compounds and initiating the biotransformation batch experiments.

### Pharmaceutical biotransformation batch experiments

The compounds selected for investigation in this study included atenolol, EE2, trimethoprim, venlafaxine, carbamazepine, glyburide and erythromycin (the compound selection process and additional information about each compound are provided in Appendix S1). Batch reactor experiments were initiated after the 14‐h regrowth period by pelleting the biomass via centrifugation and transferring the re‐suspended dilution cultures into 500 ml bottles containing SSM and pharmaceuticals at a target initial concentration of 10 μg l^−1^ each. The bottles were prepared by first drying methanol stocks containing a mixture of the compounds. Once dry, the pharmaceuticals were re‐suspended in SSM by stirring for 1 h. At each dilution level (10^−2^, 10^−4^ and 10^−7^), triplicate batch reactors with a 350‐ml starting volume were prepared. A control batch reactor was also prepared with a mixture of the biomass from each dilution level inactivated with sodium azide (0.2% w/v) (Kim *et al*., 2005). Every 24 h, an additional 2 ml of 100 g l^−1^ sodium azide stock solution was added to the control batch reactor to maintain abiotic conditions. Beginning and end‐point 20‐ml samples were collected from each batch reactor corresponding to time points of 30 min and 4 days after initiation, respectively, for pharmaceutical quantification. After collection, samples were spiked with deuterated analogs of the target compounds to achieve a target concentration of 2.5 μg l^−1^ each filtered through a 0.3‐μm glass fibre filter (Sterlitech, Kent, WA, USA) and stored at 4°C until analysis (< 24 after collection). 10 ml samples were collected at time points of approximately 30 min, 4, 8, 12, 24 and 48 h and filtered through 0.3‐μm glass fibre filter (Sterlitech) to determine dissolved organic carbon concentrations (TOC Analyzer, Shimadzu, Kyoto, Japan). Total and volatile suspended solids (TSS/VSS) concentrations were determined according to Standard methods for the examination of water and wastewater (2005) at the beginning and end of the experiment (96 h) for each batch reactor (Table S5). The average volatile suspended solids concentration between the beginning and end‐point was used to normalize all carbon oxidation rate and pharmaceutical transformation extent data (calculation details are provided in Appendix S1). Transformation extents were also normalized by the amount of time elapsed between the initial and final sample collections.

Pharmaceutical concentrations were determined via online pre‐concentration followed by high‐performance liquid chromatography and high‐resolution mass spectrometry (details in Appendix S1). Background concentrations of pharmaceuticals were considered negligible as compared to the spiked concentrations based on reported values in wastewater influents (e.g. Joss *et al*., 2005; Nakada *et al*., 2006) and previous characterization of Ann Arbor WWTP influent (Stadler *et al*., 2014). Quantification was performed using a matrix‐matched calibration curve (Fig. S1). Pharmaceutical biotransformation extents and percentage loss for each compound were calculated based on the change between the initial and final samples.

### DNA and RNA sample collection, library preparation and sequencing

Duplicate 15 ml samples from each batch reactor were collected for DNA analysis from each of the triplicate dilution cultures between 4 h 20 min and 5 h 40 min after initiating the batch experiments. The biomass was pelleted via centrifugation at 4°C and 6200 *g* for 5 min, the supernatant was discarded, and the pellet was stored at −80°C until DNA extraction. Duplicate 15 mL samples were collected for RNA analysis from each of the triplicate dilution cultures between 5 h 30 min and 7 h 50 min after initiating the batch experiments to get a representative sample of microbial activity at a time when residual organic carbon and pharmaceutical concentrations were detectable. The biomass was pelleted via centrifugation at 4°C and 6200 *g* for 5 min, the supernatant was discarded, and the pellet was re‐suspended in 2 ml of RNALater (Qiagen, Valencia, CA, USA) and stored at −80°C until RNA extraction.

DNA and RNA extractions were performed as described in the Appendix S1. Amplicon sequencing of the 16S rRNA gene and cDNA was performed on Illumina MiSeq (Illumina Inc., San Diego, CA) using universal primers F515 (5′‐ GTGCCAGCMGCCGCGGTAA‐3′) and R806 (5′‐ GGACTACHVGGGTWTCTAAT‐3′) for bacteria and archaea targeting the V4 region of the 16S rRNA gene (Kozich *et al*., 2013). DNA samples were prepared for shotgun metagenomic sequencing at the University of Michigan DNA Sequencing Core (details provided in Appendix S1) and sequenced on a 100‐cycle paired end run on a HiSeq 2500 (Illumina). RNA samples were prepared for shotgun metatranscriptomic sequencing by first enriching the mRNA from the total RNA extracts using the MICROBExpress Bacterial mRNA Enrichment Kit (Invitrogen, Carlsbad, CA) based on previous studies (He *et al*., 2010; Mettel *et al*., 2010). Individual libraries were prepared for each sample as for the DNA samples, and the samples were multiplexed using sample‐specific adaptors on a single lane of a HiSeq Flow Cell (Illumina). Sequencing analysis procedures, parameters and subsampling depths are provided in Appendix S1.

### Statistical analyses

Differences in pharmaceutical biotransformation extents and taxonomic and functional diversity measurements between the dilution conditions were tested with ANOVA. In addition, post hoc analysis using two‐sided *t*‐tests was used to perform pairwise tests of biotransformation extents and pairwise tests of taxonomic and functional diversity measurements between the different dilution conditions. Bonferroni corrections were used to account for multiple comparisons in the post hoc analyses (Abdi, 2007). We also assessed associations between the collective extents of pharmaceutical biotransformation and biodiversity by scaling each compound's normalized extent of loss (mean of 0, standard deviation of 1) (Zavaleta *et al*., 2010). Two‐sided Spearman rank correlation was used to test associations between biodiversity measurements and pharmaceutical biotransformation extents. Metatranscriptomic mapped reads were analysed using the Bioconductor DESeq2 package based on the negative binomial model (Love *et al*., 2014). More information about the statistical analysis used is given in the Appendix S1.

### Experimental data

Raw metagenomic and metatranscriptomic reads are publically available via MG‐RAST (project ID 12795). Raw 16S rRNA gene and transcript data are publically available via NCBI (project ID PRJNA319442). Assembled metagenomes are available via the Joint Genome Institute (Taxon Object ID 3300005080).

## Conflict of Interest

None declared.

## Supporting information


**Appendix S1**. Methods.
**Table S1.** Final concentrations of micronutrients in semi‐synthetic sewage media.
**Table S2**. Predicted biotransformation pathways for selected compounds. Unique pathways are indicated in bold.
**Table S3.** Target compounds with accurate mass and retention times used for quantification.
**Table S4.** Initial and final pharmaceutical concentrations in each batch reactor, biotransformation extents, and extents normalized by volatile suspended solids (VSS) concentration.
**Table S5.** Initial and final total and volatile suspended solids concentrations (TSS/VSS) in each batch reactor.
**Table S6.** List of significantly differentially expressed genes (likelihood ratio, Benjamini‐Hochberg adjusted *P* < 0.05) that were also significantly associated with compound biotransformation (Spearman, Benjamini‐Hochberg adjusted *P* < 0.05,).
**Table S7.** KEGG identifiers for functions that associated with transformation across all pharmaceutical compounds based on gene enrichment analysis.
**Fig. S1.** Standard curves prepared in SSM.
**Fig. S2.** Observed functional richness versus taxonomic richness.
**Fig. S3.** Dissolved organic carbon profiles for each batch reactor over the first 2 days of the experiment.
**Fig. S4.** RNA‐based functional richness versus scaled, normalized biotransformation extents for each compound: atenolol (a), EE2 (b), trimethoprim (c), venlafaxine (d), carbamazepine (e), glyburide (f), erythromycin (g), and collective (h).
**Fig. S5.** The relationship between the mean expression across all of the samples, and the log change in expression between the 10^−2^ and 10^−7^ cultures.
**Fig. S6**. Relative expression of significantly differentially expressed genes (likelihood ratio, Benjamini‐Hochberg adjusted *P* < 0.05) that were annotated with KO terms associated with pharmaceutical transformation based on the gene enrichment analysis.
