# Supplementary material for: Elucidating the impact of microbial community biodiversity on pharmaceutical biotransformation during wastewater treatment
Source: Microb Biotechnol. 2017 Oct 27;11(6):995–1007. doi: 10.1111/1751-7915.12870 (PMC6196385; doi:10.1111/1751-7915.12870)
Supplement: Supplementary file 1 — Appendix S1. Methods. Table S1. Final concentrations of micronutrients in semi‐synthetic sewage media. Table S2. Predicted biotransformation pathways for selected compounds. Unique pathways are indicated in bold. Table S3. Target compounds with accurate mass and retention times used for quantification. Table S4. Initial and final pharmaceutical concentrations in each batch reactor, biotransformation extents, and extents normalized by volatile suspended solids (VSS) concentration. Table S5. Initial and final total and volatile suspended solids concentrations (TSS/VSS) in each batch reactor. Table S6. List of significantly differentially expressed genes (likelihood ratio, Benjamini‐Hochberg adjusted P < 0.05) that were also significantly associated with compound biotransformation (Spearman, Benjamini‐Hochberg adjusted P < 0.05,). Table S7. KEGG identifiers for functions that associated with transformation across all pharmaceutical compounds based on gene enrichment analysis. Fig. S1. Standard curves prepared in SSM. Fig. S2. Observed functional richness versus taxonomic richness. Fig. S3. Dissolved organic carbon profiles for each batch reactor over the first 2 days of the experiment. Fig. S4. RNA‐based functional richness versus scaled, normalized biotransformation extents for each compound: atenolol (a), EE2 (b), trimethoprim (c), venlafaxine (d), carbamazepine (e), glyburide (f), erythromycin (g), and collective (h). Fig. S5. The relationship between the mean expression across all of the samples, and the log change in expression between the 10−2 and 10−7 cultures. Fig. S6. Relative expression of significantly differentially expressed genes (likelihood ratio, Benjamini‐Hochberg adjusted P < 0.05) that were annotated with KO terms associated with pharmaceutical transformation based on the gene enrichment analysis. [file MBT2-11-995-s001.docx]

# Supporting Information for

# Elucidating the impact of microbial community biodiversity on pharmaceutical biotransformation during wastewater treatment

Lauren B. Stadler^1†§^, Jeseth Delgado Vela^1†^, Sunit Jain^2∞^, Gregory J. Dick^2^, and Nancy G. Love^1*^

^1^ Department of Civil and Environmental Engineering, University of Michigan

^2^ Department of Earth and Environmental Sciences, University of Michigan

^†^ These authors contributed equally to this work

*Corresponding author:

183 EWRE, 1351 Beal Street

Ann Arbor, MI 48109, USA

Phone: 734-763-9664, Fax: 734-664-4292

E-mail: *nglove@umich.edu*

This file includes:

Pages S1 – S45

Materials and Methods: S2 –S12

Results: S12 – S43

References: S449 – S45

Number of Figures: 6

Number of Tables: 7

# *1.0 Methods*

## 1.1 Semi-synthetic sewage (SSM) media preparation

Sterile, semi-synthetic sewage media (SSM) was prepared according to the procedure outlined below. Primary effluent from the Ann Arbor wastewater treatment plant was supplemented with carbon (as humic acid, peptone, and meat extract), ammonia-N, and micronutrients. Target final concentrations of carbon and nitrogen were 1,850 mg/L as COD and 30 mg-N//L as ammonia. Humics were added such that they made up approximately 10% of the total carbon (Huang *et al.*, 2010), and the remainder of the supplemental carbon was equal parts peptone and meat extract as COD. Micronutrients were also supplemented in proportion to the COD (Grady *et al.*, 2011).

1. Freshly collected primary effluent was filtered through glass fiber filters with a pore size of 0.7 μm (Whatman GF/F, Item #0987472, Fisher Scientific, Pittsburgh, Pennsylvania)
2. Primary was then floc-filtered by adding 1 mL per 100 mL of primary of 100 g/L zinc sulfate and adjusting the pH to ~10.4 using 10 M sodium hydroxide while mixing vigorously (by placing the primary on a stir plate). After pH adjustment, mixing was halted and the flocs were allowed to settle for 3 minutes
3. The supernatant was pulled off and filtered again through a 0.2 µm filter (Stericup, Millipore, Darmstadt, Germany)
4. The filtered primary was autoclaved for 45 minutes at 121 °C.
5. After cooling, the autoclaved media was supplemented with sterile stock solutions of humic acid, meat extract and peptone, and ammonium-chloride to achieve a final concentration of 2,000 mg-COD/L and 30 mg-N/L as ammonia. Micronutrients were also supplemented assuming a yield of 0.5 g COD/g COD (Grady *et al.*, 2011) to achieve the final concentrations provided in Table S1.
6. The media was pH adjusted to 7.5 using concentrated hydrochloric acid.

**Table S1.** Final concentrations of micronutrients in semi-synthetic sewage media.

| Nutrient | Concentration (mg/L) |
| --- | --- |
| Ca | 8.2 |
| Fe | 1.65 |
| Co | 0.00033 |
| N | 71.75 |
| P | 14.35 |
| Mg | 5.77 |
| Mn | 0.08 |
| Mo | 0.0033 |
| K | 8.25 |
| S | 4.95 |
| Zn | 0.16 |
| Cu | 0.02 |
| Borate | 0.01 |

## 1.2 Compound selection approach

The primary goal of pharmaceutical compound selection was to evaluate compounds that would undergo a variety of different biochemical transformation pathways. To accomplish this we used the EAWAG-BBD Pathway Prediction System (PPS) (Ellis *et al.*, 2006; EAWAG, 2016). Using reactions found in the literature or the EAWAG’s Biocatalysis/Biodegradation and Database, PPS predicts aerobic biodegradation pathways based on chemical structure and assigns these pathways to rules. These rules are then characterized as “very likely”, “likely,” or “neutral,” by two or more biodegradation experts. We evaluated the biodegradation pathways of 22 compounds using this database accessed in May, 2014. For each compound we accounted for the “very likely”, “likely”, and “neutral” rules for the initial biotransformation step. From these 22 compounds, the list was narrowed to include compounds that could be measured in our matrix with existing methods but that still encompassed a broad range of unique rules. The final list of compounds along with the rules associated with the compounds is given in Table S2.

**Table S2**. Predicted biotransformation pathways for selected compounds. Unique pathways are indicated in bold.

| **Compound** | **Biotransformation pathways** | **Likelihood** |
| --- | --- | --- |
| Atenolol | **Primary Amide → Carboxylate** | **Likely** |
|  | primary Amine → Aldehyde or Ketone  secondary Amine → Amine + Aldehyde or Ketone  tertiary Amine →secondary Amine →Aldehyde or Ketone  Methylammonium derivative → Trimethylamine + Aldehyde or Ketone | Likely |
|  | secondary Alcohol → Ketone  secondary Alcohol →Ester | Neutral |
|  | dialiphatic Ether →Alcohol + Aldehyde  aromatic-aliphatic Ether →Phenol derivative + Aldehyde | Neutral |
| EE2 | **1-Hydroxy-2-unsubstituted aromatic → 1,2-Dihydroxyaromatic**  **4-Hydroxypyridine derivative → 3,4-Dihydroxypyridine derivative** | **Likely** |
|  | tertiary Aliphatic → tertiary Alcohol | Neutral |
|  | secondary Aliphatic →secondary Alcohol | Neutral |
| Trimethoprim | dialiphatic Ether →Alcohol + Aldehyde  aromatic-aliphatic Ether →Phenol derivative + Aldehyde | Neutral |
|  | secondary Aliphatic → secondary Alcohol | Neutral |
| Venlafaxine | primary Amine → Aldehyde or Ketone  secondary Amine → Amine + Aldehyde or Ketone  tertiary Amine →secondary Amine →Aldehyde or Ketone  Methylammonium derivative → Trimethylamine + Aldehyde or Ketone | Likely |
|  | dialiphatic Ether →Alcohol + Aldehyde  aromatic-aliphatic Ether →Phenol derivative + Aldehyde | Neutral |
|  | tertiary Aliphatic → tertiary Alcohol | Neutral |
| Carbamazepine | **N-substituted Urea derivative →Carbamate + primary Amine**  **N,N-disubstituted Urea derivative →N-substituted Carbamate + primary Amine** | **Neutral** |
| Glyburide | dialiphatic Ether →Alcohol + Aldehyde  aromatic-aliphatic Ether →Phenol derivative + Aldehyde | Neutral |
|  | **secondary Amide → Carboxylate + primary Amine**  **Lactam → Aminecarboxylate** | **Neutral** |
|  | **N-substituted Urea derivative → Carbamate + primary Amine**  **N,N-disubstituted Urea derivative → N-substituted Carbamate + primary Amine** | **Neutral** |
|  | **Sulfamate → Amine**  **Sulfonamide → Amine + Sulfonate** | **Neutral** |
|  | **N-substituted Amide → Amide + Aldehyde or Ketone**  **N, N-disubstituted Amide → N-substituted Amide + Aldehyde or Ketone**  **N-substituted Urea derivative → Urea derivative + Aldehyde or Ketone**  **N,N-disubstituted Urea derivative → N-substituted Urea derivative + Aldehyde or Ketone** | **Neutral** |
| Erythromycin | **Ester →Alcohol + Carboxylate**  **Lactone → Hydroxycarboxylate** | **Likely** |
|  | **secondary Alcohol → Ketone**  **secondary Alcohol → Ester** | **Neutral** |

## 1.3 Pharmaceutical quantification via liquid chromatography and high resolution mass spectrometry

Pharmaceuticals listed in Table S3 were quantified via on-line pre-concentration followed by high performance liquid chromatography (HPLC) and high resolution mass spectrometry (HRMS). Matrix-matched external calibration curves containing a mixture of the target compounds and deuterated analogs were used for quantification. On-line pre-concentration of the compounds of interest was performed using the Equan™ (Thermo Fisher Scientific, Grand Island, New York) system (system details are provided in Fayad et al. (2013)). The on-line pre-concentration was performed using a Hypersil Gold aQ trapping column (20 x 2.1 mm, 12 µM particle size; Thermo Fisher Scientific) and chromatographic separation was done with an Accucore aQ column (50 x 2.1 mm, 2.6 µm particle size; Thermo Fisher Scientific). A 500 µL sample was injected onto the trapping column. A mobile phase containing water with 0.1% formic acid and methanol with 0.1% formic acid was applied via gradient flow to elute all compounds with minimal overlap. The mobile phase A was water + 0.1% formic acid and B was methanol + 0.1 % formic acid. The flow rate was 0.175 mL/min and the gradient profile used was as follows: 90% A was held for 3 min, ramped to 90% of B over 8 min, held at 90% B for 1 minutes, flow rate increased to 0.25 mL/min over 0.2 minutes and held at 0.25 ml/min for 1.8 minutes, and finally returned to 90% of A and 0.175 mL/min over 0.2 min. Total run time for one sample was 16 minutes at a flow rate of 1 mL/min on the trapping column and 0.175 – 0.250 mL/min on the analytical column.

**Table S3.** Target compounds with accurate mass and retention times used for quantification.

| **Compound** | **Formula** | **Accurate mass (m/z)** | **Retention time (min)** | **ESI mode** |
| --- | --- | --- | --- | --- |
| Trimethoprim | C_14_H_18_N_4_O_3_ | 291.14516 | 8.87 | positive |
| d9-Trimethoprim | C_14_H_9_D_9_N_4_O_3_ | 300.20165 | 8.82 | positive |
| EE2 | C_20_H_24_O_2_ | 279.17434 | 13.25 | positive |
| d4-EE2 | C_20_H_20_D_4_O_2_ | 283.19944 | 13.24 | positive |
| Atenolol | C_14_H_22_N_2_O_3_ | 267.17031 | 7.41 | positive |
| d7-Atenolol | C_14_H_15_D_7_N_2_O_3_ | 274.21425 | 7.38 | positive |
| Venlafaxine | C_17_H_27_NO_2_ | 278.21145 | 10.47 | positive |
| d6-Venlafaxine | C_17_H_21_D_6_NO_2_ | 284.24911 | 10.45 | positive |
| Carbamazepine | C_15_H_12_N_2_O | 237.10223 | 12.50 | positive |
| d8-Carbamazepine | C_15_H_4_D_8_N_2_O | 245.15245 | 12.48 | positive |
| Glyburide | C_23_H_28_ClN_3_O_5_S | 494.15109 | 13.43 | positive |
| d11-Glyburide | C_23_H_17_D_11_ClN_3_O_5_S | 505.22014 | 13.41 | positive |
| Erythromycin | C_37_H_67_NO_13_ | 734.46851 | 11.44 | positive |
| d3-Erythromycin | C_37_H_64_D_3_NO_13_ | 737.48734 | 11.43 | positive |

Ionization of the compounds was achieved by positive electron spray ionization (ESI). The following source parameters were used: capillary temperature of 250 °C, auxiliary gas heater temperature of 275 °C, a spray voltage of 3.5 kV, sheath gas flow rate of 30 arbitrary units, auxiliary gas flow rate of 20 arbitrary units, and sweep gas flow rate of 1 arbitrary unit. A full scan ranging from 150 to 750 m/z was performed at a resolution of 70,000 and target automatic gain control (AGC) of 1 x 10^-6^. All data were collected and processed using the Thermo TraceFinder Software Version 3.2 (Thermo Fisher Scientific). Analytes were identified by their exact mass. Standard curves were generated by plotting the response ratio (the area of the target analyte divided by the area of the deuterated standard) versus concentration. Standard curves for the 4-day time point are given in Figure S1.

| A) | B) |
| --- | --- |
| C) | D) |
| E) | F) |
| G) |  |

**Figure S1.** Standard curves prepared in SSM. Response ratio represents the area of the analyte standard divided by the area of the deuterated standard.

## 1.4 DNA and RNA extraction

DNA and RNA extraction were performed with three bead beading steps followed by automated extraction using a Maxwell 16 automated nucleic acid extractor (Promega, Madison, WI) using the DNA blood and simplyRNA tissue kits, respectively. Extractions were conducted according to the manufacturer’s instructions except 10 μL of DNase 1 (increased from 5 μL) was used to remove contaminating DNA during RNA extractions (Webster *et al.*, 2016). Total DNA and RNA concentration in each sample were fluorometrically quantified with the Quantifluor DNA and RNA sample kits (Promega, Madison, WI). Reverse transcription to generate single-stranded complementary DNA (cDNA) from RNA extracts was performed using the SuperScript VILO cDNA Synthesis Kit according to manufacturer’s instruction (Life Technologies, Grand Island, NY).

## 1.5 Preparation of DNA and RNA for shotgun metagenomic sequencing

DNA and RNA samples were prepared for shotgun metagenomic sequencing at the University of Michigan DNA Sequencing Core. DNA was fragmented to 400 bp using standard Covaris sonication (Covaris​,​ Woburn, MA). Fragmented DNA was then prepared as a standard Illumina library using Kapa reagents (Kapa Biosystems, Wilmington MA) on an Apollo instrument (WafterGen Bio-systems, Fremont, CA), where the fragments were end-repaired, A-tailed, and adapter-ligated. The samples were then PCR amplified and pooled.​ ​ The high diversity samples (10^-2^) were sequenced on a single lane of a HiSeq Flow Cell (version 3, Illumina) and the medium (10^-4^) and low (10^-7^) diversity samples were sequenced on a second lane, with the medium diversity samples being weighted 2x the low diversity samples. The weighted pooling was chosen in order to achieve greater sequencing depth on the more diverse samples. Final libraries were checked for quality and quantity by TapeStation (Agilent, Santa Clara, CA) and qPCR using Kapa’s library quantification kit for Illumina Sequencing platforms (catalog #KK4835) (Kapa Biosystems). They were clustered on the cBot (Illumina) and​ sequenced on a 100-cycle paired end run on a HiSeq 2500 in High Output mode using version 3 reagents according to manufacturer’s protocols (Illumina). After enriching for mRNA from total RNA extracts (as described in the Materials and Methods section), individual libraries were prepared for each RNA sample as for the DNA samples and the samples were multiplexed using sample-specific adaptors on a single lane of a HiSeq Flow Cell (Illumina, Inc.). No sample-based weighting was employed for the metatranscriptomic sequencing.

## 1.6 Sequencing analysis and subsampling sequence depths

***Sequencing analysis and biodiversity measurements***

Amplicon sequencing reads were analyzed using Mothur (version 1.33.3) (Schloss *et al.*, 2009) following the MiSeq standard operating procedure (Kozich *et al.*, 2013). A copy of the batch script used for amplicon sequencing analysis using Mothur is provided below:

make.contigs(file=stability.files, processors=2)

summary.seqs(fasta=current)

screen.seqs(fasta=current, group=current, summary=current, maxambig=0, maxlength=275)

unique.seqs(fasta=current)

count.seqs(name=current, group=current)

summary.seqs(count=current)

align.seqs(fasta=current, reference=silva.v4.fasta)

summary.seqs(fasta=current, count=current)

screen.seqs(fasta=current, count=current, summary=current, start=1968, end=11550, maxhomop=8)

summary.seqs(fasta=current, count=current)

filter.seqs(fasta=current, vertical=T, trump=.)

unique.seqs(fasta=current, count=current)

pre.cluster(fasta=current, count=current, diffs=2)

chimera.uchime(fasta=current, count=current, dereplicate=t)

remove.seqs(fasta=current, accnos=current)

summary.seqs(fasta=current, count=current)

classify.seqs(fasta=current, count=current, reference=trainset9_032012.pds.fasta, taxonomy=trainset9_032012.pds.tax, cutoff=80)

remove.lineage(fasta=current, count=current, taxonomy=current, taxon=Chloroplast-Mitochondria-unknown-Eukaryota)

dist.seqs(fasta=current, cutoff=0.20)

cluster(column=current, count=current)

make.shared(list=current, count=current, label=0.03)

classify.otu(list=current, count=current, taxonomy=current, label=0.03)

count.groups(shared=current)

Sequences were binned into OTUs based on sequence similarity of greater or equal to 97%. Taxonomic biodiversity measurements were calculated based on OTUs. 16S rRNA gene and 16S rRNA sequencing data were analyzed using Mothur and differences in sequencing depth were corrected for by subsampling to the lowest number of sequences per sample for all samples (19,681 for DNA, and 29,428 for cDNA). Sequence classification was performed using the 16S rRNA gene taxonomy from the Ribosomal Database Project (Cole *et al.*, 2009). Sequences were deposited on NCBI (project ID PRJNA319442).

Raw shotgun sequencing reads were dereplicated (100% identity over 100% of the length) and trimmed using Sickle (Joshi and Fass, 2011). For the trimming of the raw shotgun sequencing reads, adaptors were removed using Scythe version 0.993b (https://github.com/Geo-omics/scripts/tree/master/DerepTools). Trimming removed 8-19% of the data. Whole genome de novo assembly was performed by pooling all reads from the dilution cultures and using IDBA-UD (Peng *et al.*, 2012). Assembly was performed using the following parameters: mink 52, maxk 93, step 8. Assembled data resulted in 910, 247 contigs (of which 110,766 contigs were > 1,000 bp). The N50 of the assembled contigs was 1,645 bp. Because of the low N50 and relatively short average contig length, we chose to take a gene-centric analysis approach (as opposed to a genome-centric approach), as assembly of genomes was not feasible with such short contigs. Assembled data was submitted to the DOE JGI-IMG/MER annotation pipeline (Taxon Object ID 3300005080). The assembled contigs were annotated using KEGG Orthology (KO) database in IMG. KEGG Orthology (KO) annotations from the IMG analysis were screened to retain those above a threshold of an e-value less than 10^-5^, bit score greater than 50, and percent identity greater than 60%. Paired-end transcriptomic reads were mapped to assembled contigs using the Burrows-Wheeler Aligner (BWA version 0.7.10) using the default parameters (Li and Durbin, 2009). For the transcriptomic read analysis, paired forward and reverse read alignments were generated in the SAM format using BWASAMPE algorithm. The mapped read counts were extracted using SAMtools (Li *et al.*, 2009). When aligning transcriptomic reads to the contigs using the BWA default parameters, only the reads with at most 1 mismatch to the region of the contig was allowed. Furthermore, only alignments with an overall score greater than 30 were considered. Each replicate was mapped to the pooled assembly separately. HTSeq count was used to obtain the raw counts per gene for each sample, which was used for all subsequent analyses (Anders *et al.*, 2014). Functional diversity metrics were calculating after subsampling to the lowest number of sequences per sample (1,233,846 and 624,845 for the metagenomic and metatranscriptomic reads, respectively) to correct for differences in sequencing depth.

## 1.7 Statistical Analysis

Statistical analyses were performed in the R environment (Venables *et al.*, 2004) using the stats and vegan (Oksanen *et al.*, 2007) packages. In correlations between pharmaceutical biotransformation and RNA markers (metatranscriptomic reads and 16S rRNA data), Spearman’s rank correlation was conducted using all nine data points. All nine data points were assumed to be independent because the biotransformation in each batch culture was independent as pharmaceutical compounds were added and measured in each individual batch.

For differential expression analysis using the established Bioconductor DESeq2 package (Love *et al.*, 2014), raw counts were normalized using relative library sizes, and significant differential expression was tested using the likelihood ratio test. The normalization procedure used in the DESeq2 package corrects counts by normalizing to the average expression across all the samples, each gene is normalized separately and gene-wise dispersion estimates based on a maximum likelihood estimation. In this package, the logarithmic fold changes utilize a semi empirical Bayes approach. The likelihood ratio test is a hypothesis test assuming that RNA sequencing data fit a negative binomial distribution; it is used on each gene to estimate whether the logarithmic fold changes observed are significantly different from zero. The association between the normalized expression of each gene and biotransformation extent of each pharmaceutical was tested using a two-sided Spearman rank correlation. We used the Benjamini-Hochberg method to correct for multiple comparisons (Benjamini and Hochberg, 1995). Only genes with more than one count (i.e. a raw read normalized by relative library size) across the samples were included in the analysis. Gene set enrichment analysis was conducted using the R Package clusterProfiler (Release 3.4) (Yu *et al.*, 2012) to identify gene families with expression profiles that associated with transformation across all of the compounds.

# *2.0 Results*

## 2.1 Taxonomic and functional richness of dilution cultures

We tested whether taxonomic richness positively associated with functional richness across all the conditions. We found a positive and significant association between DNA-based taxonomic richness and DNA-based functional richness (Spearman, *P*=0.0045, ρ=0.87), as well as between RNA-based taxonomic richness and RNA-based functional richness (Spearman, *P*=0.00035, ρ=0.95) (Figure S2).

**Figure S2.** Observed functional richness versus taxonomic richness. DNA-based annotations are shown in filled markers and RNA-based annotations in open markers. 10^-2^, 10^-4^, and 10^-7^ samples are represented by black, dark grey, and light grey markers, respectively. Reported *P*-values and ρ (rho; Spearman rank correlation coefficient) are based on a two-sided Spearman rank correlation test and indicate significant positive association between taxonomic and functional richness for both DNA and RNA-based annotations.

## 2.2 Carbon oxidation rate

Dissolved organic carbon was measured throughout the batch experiments. Carbon oxidation rates were determined by finding the slope of the line of the linear curve fit to the total dissolved carbon ln(C/C_0_) versus time data (Figure S2). We found no differences in the rate of carbon oxidation in the dilution cultures (Figure S2). Dissolved organic carbon remained constant throughout the control batch experiment, indicating that the sodium azide supplemented to the control served to sufficiently inactivate carbon oxidation activity throughout the experiment.


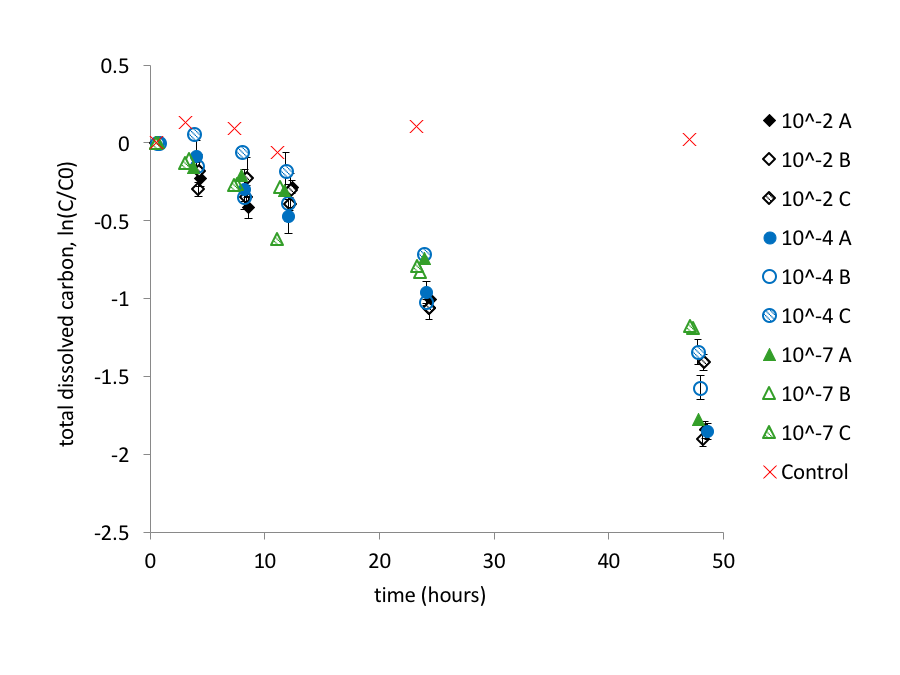


**Figure S3.** Dissolved organic carbon profiles for each batch reactor over the first 2 days of the experiment. Samples from the 10^-2^ condition are shown in black, 10^-4^ in blue, 10^-7^ in green, and then control in red. Error bars represent the standard deviation of technical triplicates

## 2.3 Pharmaceutical biotransformation data

Pharmaceutical biotransformation extents were calculated using the initial and final concentrations of the pharmaceuticals in each of the batch reactors and normalized by the exact amount of time between initial and final sample collections. Initial and final concentrations of the pharmaceuticals, calculated biotransformation extents, and normalized biotransformation extents are shown in Table S4. Extents were also normalized using the average of the initial (t ~ 0 days) and final (t ~ 4 days) volatile suspended solids concentration of the batch reactors (Table S5). Biotransformation extents for each compound in each condition were scaled such that the average of the extents were 0 and the standard deviation was equal to 1. All compound concentrations, except for atenolol from the 10^-2^ condition at the 4-d time point, were above the lowest concentration in the standard curve (0.5 μg/L). Atenolol was not detectable in the final time point in the 10^-2^ condition. Thus, it is possible that the calculated biotransformation extent is either (1) an overestimation because the concentration of atenolol was actually nonzero, but below 0.5 μg/L; or (2) and underestimation because atenolol was depleted well before the final sample. As we do not have sufficient data to address this, we assumed atenolol was equal to zero in the 10^-2^ condition and calculated the extent using that assumption. The collective biotransformation extent was calculated by averaging the scaled biotransformation extents (Zavaleta *et al.*, 2010). Figure S4 shows the scaled biotransformation extent for each individual compound (a-g) and the collective extent (h) versus RNA-based functional richness and 16S rRNA transcript richness, or the expressed taxonomic richness.

**Table S4.** Initial and final pharmaceutical concentrations in each batch reactor, biotransformation extents, and extents normalized by volatile suspended solids (VSS) concentration. Exact samples times were used to calculate the reported extents.

|  | **Concentration (µg/L)** | | **Extent** | **Extent/VSS** |
| --- | --- | --- | --- | --- |
|  | **initial** | **final** | **(µg/L/d)** | **(1/d)** |
| **atenolol** |  |  |  |  |
| 10^-2 A | 9.96 | 0.00 | 2.47 | 1.10E-05 |
| 10^-2 B | 9.64 | 0.00 | 2.40 | 1.04E-05 |
| 10^-2 C | 9.44 | 0.00 | 2.35 | 1.14E-05 |
| 10^-4 A | 9.29 | 5.39 | 0.98 | 6.01E-06 |
| 10^-4 B | 9.60 | 5.23 | 1.09 | 8.28E-06 |
| 10^-4 C | 9.19 | 4.07 | 1.28 | 8.59E-06 |
| 10^-7 A | 9.42 | 8.04 | 0.34 | 2.11E-06 |
| 10^-7 B | 9.87 | 7.53 | 0.59 | 2.74E-06 |
| 10^-7 C | 9.37 | 8.92 | 0.11 | 5.02E-07 |
| Control | 9.80 | 9.26 | 0.13 | 1.09E-06 |
| **EE2** |  |  |  |  |
| 10^-2 A | 8.26 | 4.08 | 1.04 | 4.62E-06 |
| 10^-2 B | 8.03 | 3.65 | 1.09 | 4.71E-06 |
| 10^-2 C | 8.08 | 4.32 | 0.94 | 4.53E-06 |
| 10^-4 A | 7.36 | 7.02 | 0.08 | 5.21E-07 |
| 10^-4 B | 7.36 | 7.20 | 0.04 | 2.99E-07 |
| 10^-4 C | 7.99 | 6.92 | 0.27 | 1.79E-06 |
| 10^-7 A | 8.16 | 7.28 | 0.22 | 1.35E-06 |
| 10^-7 B | 8.12 | 6.87 | 0.31 | 1.47E-06 |
| 10^-7 C | 7.40 | 8.15 | -0.19 | -8.27E-07 |
| Control | 8.03 | 9.62 | -0.40 | -3.21E-06 |
| **trimethoprim** | |  |  |  |
| 10^-2 A | 8.66 | 8.21 | 0.11 | 4.97E-07 |
| 10^-2 B | 9.28 | 8.31 | 0.24 | 1.04E-06 |
| 10^-2 C | 9.37 | 8.40 | 0.24 | 1.17E-06 |
| 10^-4 A | 8.84 | 8.99 | -0.04 | -2.25E-07 |
| 10^-4 B | 9.00 | 8.87 | 0.03 | 2.44E-07 |
| 10^-4 C | 8.31 | 9.05 | -0.19 | -1.25E-06 |
| 10^-7 A | 9.67 | 9.53 | 0.03 | 2.13E-07 |
| 10^-7 B | 8.64 | 9.78 | -0.29 | -1.34E-06 |
| 10^-7 C | 9.64 | 9.98 | -0.08 | -3.71E-07 |
| Control | 8.80 | 9.18 | -0.10 | -7.66E-07 |
| **venlafaxine** | |  |  |  |
| 10^-2 A | 9.33 | 8.17 | 0.29 | 1.28E-06 |
| 10^-2 B | 9.11 | 8.38 | 0.18 | 7.89E-07 |
| 10^-2 C | 9.62 | 8.68 | 0.23 | 1.13E-06 |
| 10^-4 A | 9.20 | 8.87 | 0.08 | 5.06E-07 |
| 10^-4 B | 9.13 | 8.75 | 0.09 | 7.18E-07 |
| 10^-4 C | 9.48 | 9.02 | 0.12 | 7.75E-07 |
| 10^-7 A | 9.19 | 8.99 | 0.05 | 3.21E-07 |
| 10^-7 B | 9.09 | 8.67 | 0.11 | 4.92E-07 |
| 10^-7 C | 9.27 | 9.04 | 0.06 | 2.48E-07 |
| Control | 9.18 | 10.13 | -0.24 | -1.92E-06 |
| **carbamazepine** | |  |  |  |
| 10^-2 A | 10.11 | 9.59 | 0.13 | 5.67E-07 |
| 10^-2 B | 11.22 | 8.52 | 0.67 | 2.90E-06 |
| 10^-2 C | 10.49 | 8.19 | 0.57 | 2.76E-06 |
| 10^-4 A | 10.32 | 11.66 | -0.33 | -2.06E-06 |
| 10^-4 B | 11.71 | 11.83 | -0.03 | -2.31E-07 |
| 10^-4 C | 11.56 | 14.98 | -0.85 | -5.74E-06 |
| 10^-7 A | 11.70 | 15.66 | -0.99 | -6.08E-06 |
| 10^-7 B | 12.73 | 16.22 | -0.88 | -4.09E-06 |
| 10^-7 C | 13.15 | 18.93 | -1.45 | -6.36E-06 |
| Control | 11.55 | 13.02 | -0.37 | -2.98E-06 |
| **glyburide** |  |  |  |  |
| 10^-2 A | 9.51 | 9.64 | -0.03 | -1.37E-07 |
| 10^-2 B | 8.99 | 9.36 | -0.09 | -4.02E-07 |
| 10^-2 C | 9.61 | 9.42 | 0.05 | 2.30E-07 |
| 10^-4 A | 9.41 | 9.25 | 0.04 | 2.48E-07 |
| 10^-4 B | 9.59 | 9.33 | 0.07 | 4.95E-07 |
| 10^-4 C | 9.13 | 9.74 | -0.15 | -1.03E-06 |
| 10^-7 A | 9.60 | 9.71 | -0.03 | -1.78E-07 |
| 10^-7 B | 9.66 | 9.77 | -0.03 | -1.33E-07 |
| 10^-7 C | 9.70 | 10.57 | -0.22 | -9.64E-07 |
| Control | 9.80 | 9.99 | -0.05 | -3.83E-07 |
| **erythromycin** | |  |  |  |
| 10^-2 A | 9.52 | 3.97 | 1.38 | 6.13E-06 |
| 10^-2 B | 9.10 | 4.34 | 1.18 | 5.12E-06 |
| 10^-2 C | 9.74 | 3.97 | 1.43 | 6.95E-06 |
| 10^-4 B | 9.36 | 2.43 | 1.73 | 1.31E-05 |
| 10^-4 C | 9.62 | 4.19 | 1.36 | 9.11E-06 |
| 10^-7 A | 10.12 | 6.18 | 0.98 | 6.04E-06 |
| 10^-7 B | 9.46 | 5.05 | 1.11 | 5.17E-06 |
| 10^-7 C | 9.18 | 5.17 | 1.01 | 4.40E-06 |
| Control | 9.83 | 9.09 | 0.19 | 1.51E-06 |

**Table S5.** Initial and final total and volatile suspended solids concentrations (TSS/VSS) in each batch reactor. Error reported for the t = 0 and 4 day measurements is the range of duplicate measurements. Average VSS concentrations were used to normalize biotransformation extent data.

| **Batch condition** | **TSS, t=0 d (mg/L)** | **TSS, t=4 d (mg/L)** | **VSS, t=0 d (mg/L)** | **VSS, t=4 d (mg/L)** | **TSS, average (0, 4d; mg/L)** | **VSS, average (0, 4d; mg/L)** |
| --- | --- | --- | --- | --- | --- | --- |
| 10^-2 A | 196 ± 11 | 337 ± 5 | 169 ± 6 | 281 ± 8 | 266 ± 12 | 225 ± 10 |
| 10^-2 B | 190 ± 5 | 361 ± 64 | 164 ± 8 | 298 ± 40 | 275 ± 64 | 231 ± 41 |
| 10^-2 C | 195 ± 0 | 287 ± 18 | 165 ± 0 | 248 ± 3 | 241 ± 18 | 207 ± 3 |
| 10^-4 A | 113 ± 5 | 256 ± 5 | 98 ± 5 | 227 ± 9 | 184 ± 7 | 162 ± 10 |
| 10^-4 B | 103 ± 6 | 208 ± 4 | 88 ± 0 | 176 ± 5 | 156 ± 7 | 132 ± 5 |
| 10^-4 C | 96 ± 12 | 249 ± 5 | 80 ± 8 | 218 ± 0 | 173 ± 14 | 149 ± 8 |
| 10^-7 A | 84 ± 5 | 290 ± 14 | 72 ± 4 | 255 ± 13 | 187 ± 15 | 163 ± 14 |
| 10^-7 B | 93 ± 13 | 387 ± 131 | 80 ± 11 | 348 ± 110 | 240 ± 131 | 214 ± 111 |
| 10^-7 C | 106 ± 7 | 412 ± 17 | 90 ± 10 | 367 ± 0 | 259 ± 18 | 229 ± 10 |
| control | 196 ± 25 | 123 ± 1 | 167 ± 20 | 81 ± 1 | 159 ± 25 | 124 ± 20 |

Expressed taxonomic richness

RNA-based functional richness

**Figure S4.** RNA-based functional richness versus scaled, normalized biotransformation extents for each compound: atenolol (a), EE2 (b), trimethoprim (c), venlafaxine (d), carbamazepine (e), glyburide (f), erythromycin (g), and collective (h). Orange circles indicate the RNA-based functional richness, on the scale on the left. Black X’s indicate the average transformation extent and average RNA-based functional richness at each dilution condition. Blue triangles indicate the expressed taxonomic richness (based on the richness of the 16S rRNA transcript), shown on scale on the right. Black horizontal lines indicate the average transformation extent and average expressed taxonomic richness at each dilution condition. A scaled biotransformation extent value of zero indicates the average extent of biotransformation for all the dilution conditions.

##
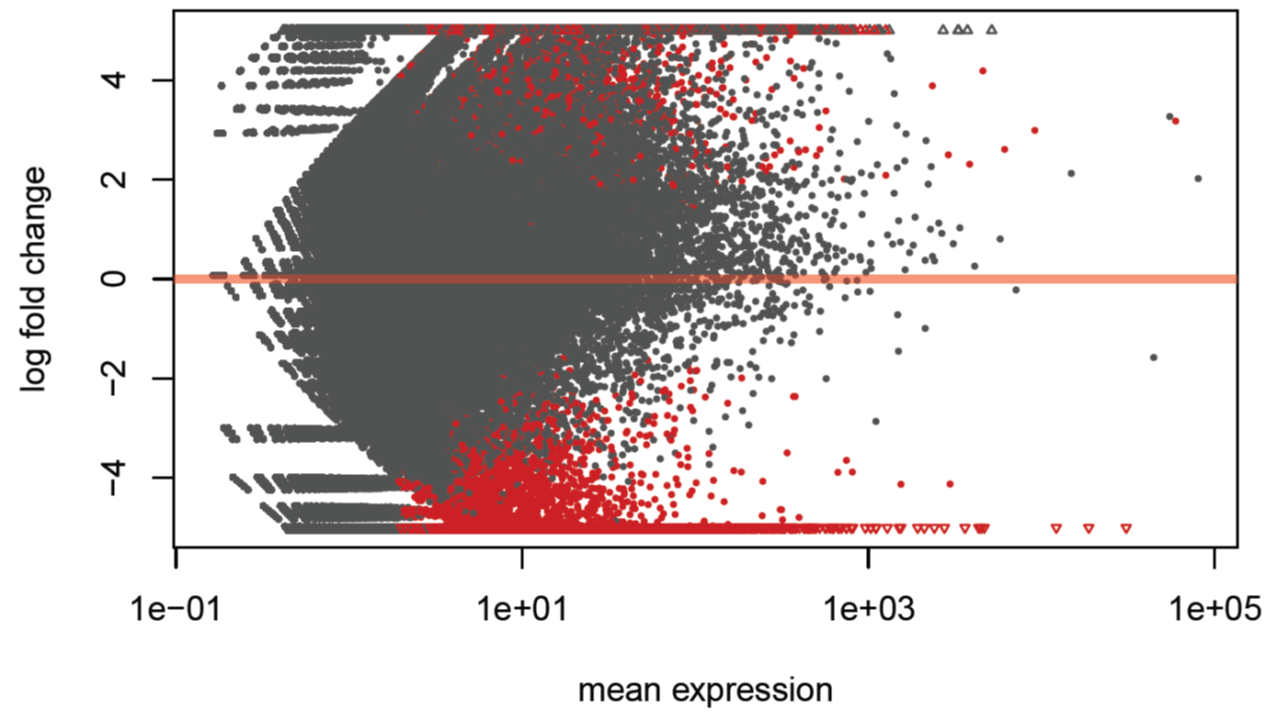


**Figure S5.** The relationship between the mean expression across all of the samples, and the log change in expression between the 10^-2^ and 10^-7^ cultures. Each gene is shown in grey. Statistically significant (likelihood ratio, Benjamini-Hochberg adjusted *P*<0.05) differentially expressed genes are shown in red.

## 2.4 Candidate gene lists for each compound

To identify genes whose expression pattern was significant associated with biotransformation extents we first narrowed the list of genes by focusing on classes of metabolic genes that were predicted, according to the PPS (Ellis *et al.*, 2006; EAWAG, 2016), to be involved in the compounds biotransformation. We then conducted a two-sided Spearman’s rank correlation test between the normalized expression of each gene at each dilution condition and the biotransformation extent in each batch for each compound. We focused on the compounds that were transformed to different extents with increased dilution (atenolol, EE2, trimethoprim, venlafaxine, and carbamazepine) and further narrowed the list of genes relevant to those compounds by selecting genes only if they were lost with increased dilution. We also performed a two-sided Spearman’s rank correlation test for erythromycin, but did not narrow the genes to those lost during dilution. A complete list of candidate genes for each compound is provided below (Table S6).

**Table S6.** List of significantly differentially expressed genes (likelihood ratio, Benjamini-Hochberg adjusted *P* < 0.05) that were also significantly associated with compound biotransformation (Spearman, Benjamini-Hochberg adjusted *P*<0.05,). Bolded KEGG orthology numbers had more than one significant gene mapped to the function; statistical values represent an average of all the genes which mapped to the KEGG number.

| **Atenolol** | | | |
| --- | --- | --- | --- |
| **KEGG Orthology number** | **Description** | ***P*-value** | **Spearman’s ρ** |
| K00001 | alcohol dehydrogenase [EC:1.1.1.1] | 0.037 | 0.746 |
| **K00003** | **homoserine dehydrogenase [EC:1.1.1.3]** | **0.028** | **0.788** |
| K00005 | glycerol dehydrogenase [EC:1.1.1.6] | 0.001 | 0.949 |
| **K00010** | **myo-inositol 2-dehydrogenase [EC:1.1.1.18]** | **0.022** | **0.814** |
| **K00012** | **UDPglucose 6-dehydrogenase [EC:1.1.1.22]** | **0.026** | **0.783** |
| **K00013** | **histidinol dehydrogenase [EC:1.1.1.23]** | **0.021** | **0.805** |
| **K00014** | **shikimate dehydrogenase [EC:1.1.1.25];shikimate 5-dehydrogenase [EC:1.1.1.25]** | **0.006** | **0.898** |
| K00016 | L-lactate dehydrogenase [EC:1.1.1.27] | 0.044 | 0.729 |
| K00018 | glycerate dehydrogenase [EC:1.1.1.29] | 0.003 | 0.915 |
| K00019 | 3-hydroxybutyrate dehydrogenase [EC:1.1.1.30] | 0.005 | 0.898 |
| **K00020** | **3-hydroxyisobutyrate dehydrogenase [EC:1.1.1.31]** | **0.015** | **0.854** |
| **K00024** | **malate dehydrogenase [EC:1.1.1.37]** | **0.009** | **0.888** |
| **K00027** | **malate dehydrogenase (oxaloacetate-decarboxylating) [EC:1.1.1.38]** | **0.027** | **0.788** |
| **K00029** | **malate dehydrogenase (oxaloacetate-decarboxylating)(NADP+) [EC:1.1.1.40]** | **0.013** | **0.857** |
| **K00031** | **isocitrate dehydrogenase [EC:1.1.1.42]** | **0.014** | **0.849** |
| K00052 | 3-isopropylmalate dehydrogenase [EC:1.1.1.85] | 0.014 | 0.831 |
| K00055 | aryl-alcohol dehydrogenase [EC:1.1.1.90] | 0.018 | 0.814 |
| **K00057** | **glycerol-3-phosphate dehydrogenase (NAD(P)+) [EC:1.1.1.94]** | **0.012** | **0.860** |
| **K00058** | **D-3-phosphoglycerate dehydrogenase [EC:1.1.1.95]** | **0.018** | **0.822** |
| **K00074** | **3-hydroxybutyryl-CoA dehydrogenase [EC:1.1.1.157]** | **0.029** | **0.777** |
| K00075 | UDP-N-acetylmuramate dehydrogenase [EC:1.1.1.158] | 0.026 | 0.780 |
| **K00088** | **IMP dehydrogenase [EC:1.1.1.205]** | **0.012** | **0.850** |
| K00102 | D-lactate dehydrogenase (cytochrome) [EC:1.1.2.4] | 0.003 | 0.915 |
| **K00111** | **glycerol-3-phosphate dehydrogenase [EC:1.1.5.3]** | **0.013** | **0.867** |
| **K00116** | **malate dehydrogenase (quinone) [EC:1.1.5.4]** | **0.012** | **0.867** |
| **K00117** | **quinoprotein glucose dehydrogenase [EC:1.1.5.2]** | **0.022** | **0.800** |
| **K00121** | **S-(hydroxymethyl)glutathione dehydrogenase / alcohol dehydrogenase [EC:1.1.1.284 1.1.1.1]** | **0.013** | **0.844** |
| **K00123** | **formate dehydrogenase, alpha subunit [EC:1.2.1.2]** | **0.009** | **0.891** |
| **K00124** | **formate dehydrogenase, beta subunit [EC:1.2.1.2]** | **0.006** | **0.907** |
| **K00127** | **formate dehydrogenase, gamma subunit [EC:1.2.1.2]** | **0.004** | **0.909** |
| **K00128** | **aldehyde dehydrogenase (NAD+) [EC:1.2.1.3]** | **0.009** | **0.866** |
| K00130 | betaine-aldehyde dehydrogenase [EC:1.2.1.8] | 0.005 | 0.898 |
| **K00133** | **aspartate-semialdehyde dehydrogenase [EC:1.2.1.11]** | **0.007** | **0.892** |
| **K00134** | **glyceraldehyde 3-phosphate dehydrogenase [EC:1.2.1.12]** | **0.014** | **0.855** |
| **K00135** | **succinate-semialdehyde dehydrogenase (NADP+) [EC:1.2.1.16]** | **0.017** | **0.845** |
| **K00140** | **malonate-semialdehyde dehydrogenase (acetylating) / methylmalonate-semialdehyde dehydrogenase [EC:1.2.1.18 1.2.1.27];methylmalonate-semialdehyde dehydrogenase [EC:1.2.1.27]** | **0.013** | **0.859** |
| **K00146** | **phenylacetaldehyde dehydrogenase [EC:1.2.1.39]** | **0.006** | **0.881** |
| **K00147** | **glutamate-5-semialdehyde dehydrogenase [EC:1.2.1.41]** | **0.007** | **0.886** |
| K00156 | pyruvate dehydrogenase (quinone) [EC:1.2.5.1];pyruvate dehydrogenase (cytochrome) [EC:1.2.2.2] | 0.022 | 0.797 |
| **K00161** | **pyruvate dehydrogenase E1 component subunit alpha [EC:1.2.4.1]** | **0.012** | **0.864** |
| K00162 | pyruvate dehydrogenase E1 component subunit beta [EC:1.2.4.1] | 0.031 | 0.763 |
| **K00163** | **pyruvate dehydrogenase E1 component [EC:1.2.4.1]** | **0.009** | **0.887** |
| **K00164** | **2-oxoglutarate dehydrogenase E1 component [EC:1.2.4.2]** | **0.009** | **0.879** |
| **K00166** | **2-oxoisovalerate dehydrogenase E1 component, alpha subunit [EC:1.2.4.4]** | **0.006** | **0.881** |
| K00228 | coproporphyrinogen III oxidase [EC:1.3.3.3] | 0.003 | 0.915 |
| K00239 | succinate dehydrogenase flavoprotein subunit [EC:1.3.99.1] | 0.011 | 0.863 |
| **K00240** | **succinate dehydrogenase iron-sulfur protein [EC:1.3.99.1];succinate dehydrogenase iron-sulfur subunit [EC:1.3.99.1]** | **0.014** | **0.862** |
| **K00241** | **succinate dehydrogenase cytochrome b556 subunit;succinate dehydrogenase cytochrome b-556 subunit [EC:1.3.99.1];succinate dehydrogenase cytochrome b-556 subunit** | **0.015** | **0.849** |
| K00242 | succinate dehydrogenase hydrophobic membrane anchor protein [EC:1.3.99.1];succinate dehydrogenase hydrophobic membrane anchor protein;succinate dehydrogenase membrane anchor subunit | 0.044 | 0.729 |
| K00249 | acyl-CoA dehydrogenase [EC:1.3.99.3] | 0.017 | 0.818 |
| **K00252** | **glutaryl-CoA dehydrogenase [EC:1.3.99.7]** | **0.010** | **0.870** |
| K00253 | isovaleryl-CoA dehydrogenase [EC:1.3.99.10] | 0.003 | 0.915 |
| **K00254** | **dihydroorotate dehydrogenase [EC:1.3.5.2]** | **0.023** | **0.805** |
| K00255 | long-chain-acyl-CoA dehydrogenase [EC:1.3.99.13] | 0.006 | 0.881 |
| **K00259** | **alanine dehydrogenase [EC:1.4.1.1]** | **0.010** | **0.883** |
| **K00261** | **glutamate dehydrogenase (NAD(P)+) [EC:1.4.1.3]** | **0.008** | **0.876** |
| **K00262** | **glutamate dehydrogenase (NADP+) [EC:1.4.1.4]** | **0.012** | **0.849** |
| **K00263** | **leucine dehydrogenase [EC:1.4.1.9]** | **0.004** | **0.907** |
| K00274 | monoamine oxidase [EC:1.4.3.4] | 0.001 | 0.949 |
| **K00281** | **glycine dehydrogenase [EC:1.4.4.2]** | **0.009** | **0.885** |
| **K00285** | **D-amino-acid dehydrogenase [EC:1.4.99.1]** | **0.013** | **0.855** |
| **K00294** | **1-pyrroline-5-carboxylate dehydrogenase [EC:1.5.1.12]** | **0.028** | **0.799** |
| **K00318** | **proline dehydrogenase [EC:1.5.99.8]** | **0.020** | **0.814** |
| **K00330** | **NADH-quinone oxidoreductase subunit A [EC:1.6.5.3];NADH dehydrogenase I subunit A [EC:1.6.5.3]** | **0.009** | **0.874** |
| **K00331** | **NADH dehydrogenase I subunit B [EC:1.6.5.3];NADH-quinone oxidoreductase subunit B [EC:1.6.5.3]** | **0.014** | **0.837** |
| **K00332** | **NADH-quinone oxidoreductase subunit C [EC:1.6.5.3];NADH dehydrogenase I subunit C [EC:1.6.5.3]** | **0.004** | **0.911** |
| **K00333** | **NADH dehydrogenase I subunit D [EC:1.6.5.3];NADH-quinone oxidoreductase subunit D [EC:1.6.5.3]** | **0.006** | **0.906** |
| **K00334** | **NADH-quinone oxidoreductase subunit E [EC:1.6.5.3];NADH dehydrogenase I subunit E [EC:1.6.5.3]** | **0.012** | **0.870** |
| **K00335** | **NADH dehydrogenase I subunit F [EC:1.6.5.3];NADH-quinone oxidoreductase subunit F [EC:1.6.5.3]** | **0.009** | **0.874** |
| **K00336** | **NADH-quinone oxidoreductase subunit G [EC:1.6.5.3];NADH dehydrogenase I subunit G [EC:1.6.5.3]** | **0.012** | **0.863** |
| **K00337** | **NADH dehydrogenase I subunit H [EC:1.6.5.3];NADH-quinone oxidoreductase subunit H [EC:1.6.5.3]** | **0.016** | **0.836** |
| **K00338** | **NADH-quinone oxidoreductase subunit I [EC:1.6.5.3];NADH dehydrogenase I subunit I [EC:1.6.5.3]** | **0.016** | **0.850** |
| **K00339** | **NADH dehydrogenase I subunit J [EC:1.6.5.3];NADH-quinone oxidoreductase subunit J [EC:1.6.5.3]** | **0.008** | **0.881** |
| **K00340** | **NADH-quinone oxidoreductase subunit K [EC:1.6.5.3];NADH dehydrogenase I subunit K [EC:1.6.5.3]** | **0.022** | **0.797** |
| **K00341** | **NADH dehydrogenase I subunit L [EC:1.6.5.3];NADH-quinone oxidoreductase subunit L [EC:1.6.5.3]** | **0.010** | **0.870** |
| **K00342** | **NADH-quinone oxidoreductase subunit M [EC:1.6.5.3];NADH dehydrogenase I subunit M [EC:1.6.5.3]** | **0.017** | **0.835** |
| **K00343** | **NADH dehydrogenase I subunit N [EC:1.6.5.3];NADH-quinone oxidoreductase subunit N [EC:1.6.5.3]** | **0.017** | **0.831** |
| **K00382** | **dihydrolipoamide dehydrogenase [EC:1.8.1.4]** | **0.013** | **0.864** |
| **K00404** | **cb-type cytochrome c oxidase subunit I [EC:1.9.3.1];cytochrome c oxidase cb-type subunit I [EC:1.9.3.1]** | **0.008** | **0.886** |
| **K00405** | **cb-type cytochrome c oxidase subunit II [EC:1.9.3.1];cytochrome c oxidase cb-type subunit II** | **0.005** | **0.904** |
| **K00406** | **cb-type cytochrome c oxidase subunit III [EC:1.9.3.1];cytochrome c oxidase cb-type subunit III** | **0.005** | **0.911** |
| **K00425** | **cytochrome d ubiquinol oxidase subunit I [EC:1.10.3.-];cytochrome bd-I oxidase subunit I [EC:1.10.3.-]** | **0.013** | **0.852** |
| **K00426** | **cytochrome d ubiquinol oxidase subunit II [EC:1.10.3.-];cytochrome bd-I oxidase subunit II [EC:1.10.3.-]** | **0.016** | **0.846** |
| **K00432** | **glutathione peroxidase [EC:1.11.1.9]** | **0.020** | **0.817** |
| **K00457** | **4-hydroxyphenylpyruvate dioxygenase [EC:1.13.11.27]** | **0.013** | **0.862** |
| **K00459** | **nitronate monooxygenase [EC:1.13.12.16]** | **0.007** | **0.885** |
| K00466 | tryptophan 2-monooxygenase [EC:1.13.12.3] | 0.003 | 0.915 |
| **K00499** | **choline monooxygenase [EC:1.14.15.7]** | **0.019** | **0.831** |
| **K00500** | **phenylalanine-4-hydroxylase [EC:1.14.16.1]** | **0.010** | **0.887** |
| **K00602** | **phosphoribosylaminoimidazolecarboxamide formyltransferase / IMP cyclohydrolase [EC:2.1.2.3 3.5.4.10]** | **0.016** | **0.847** |
| **K00627** | **pyruvate dehydrogenase E2 component (dihydrolipoamide acetyltransferase) [EC:2.3.1.12]** | **0.021** | **0.815** |
| **K00658** | **2-oxoglutarate dehydrogenase E2 component (dihydrolipoamide succinyltransferase) [EC:2.3.1.61]** | **0.014** | **0.850** |
| **K00812** | **aspartate aminotransferase [EC:2.6.1.1]** | **0.019** | **0.828** |
| **K00817** | **histidinol-phosphate aminotransferase [EC:2.6.1.9]** | **0.013** | **0.859** |
| **K00818** | **acetylornithine aminotransferase [EC:2.6.1.11]** | **0.006** | **0.898** |
| **K00820** | **glucosamine--fructose-6-phosphate aminotransferase (isomerizing) [EC:2.6.1.16]** | **0.024** | **0.792** |
| **K00821** | **acetylornithine/N-succinyldiaminopimelate aminotransferase [EC:2.6.1.11 2.6.1.17]** | **0.007** | **0.878** |
| **K00822** | **beta-alanine--pyruvate transaminase [EC:2.6.1.18]** | **0.005** | **0.924** |
| **K00823** | **4-aminobutyrate aminotransferase [EC:2.6.1.19]** | **0.005** | **0.908** |
| **K00826** | **branched-chain amino acid aminotransferase [EC:2.6.1.42]** | **0.014** | **0.844** |
| **K00831** | **phosphoserine aminotransferase [EC:2.6.1.52]** | **0.020** | **0.824** |
| **K00832** | **aromatic-amino-acid transaminase [EC:2.6.1.57]** | **0.029** | **0.771** |
| **K00836** | **diaminobutyrate-2-oxoglutarate transaminase [EC:2.6.1.76]** | **0.022** | **0.805** |
| K01066 | esterase / lipase [EC:3.1.1.-] | 0.044 | 0.729 |
| **K01067** | **acetyl-CoA hydrolase [EC:3.1.2.1]** | **0.023** | **0.805** |
| **K01139** | **guanosine-3',5'-bis(diphosphate) 3'-pyrophosphohydrolase [EC:3.1.7.2]** | **0.017** | **0.826** |
| **K01426** | **amidase [EC:3.5.1.4]** | **0.020** | **0.805** |
| K01434 | penicillin amidase [EC:3.5.1.11] | 0.026 | 0.780 |
| K01436 | aminoacylase [EC:3.5.1.14];amidohydrolase [EC:3.5.1.-] | 0.005 | 0.898 |
| **K01442** | **choloylglycine hydrolase [EC:3.5.1.24]** | **0.015** | **0.856** |
| **K01484** | **succinylarginine dihydrolase [EC:3.5.3.23]** | **0.013** | **0.881** |
| **K01491** | **methylenetetrahydrofolate dehydrogenase (NADP+) / methenyltetrahydrofolate cyclohydrolase [EC:1.5.1.5 3.5.4.9]** | **0.005** | **0.893** |
| **K01495** | **GTP cyclohydrolase I [EC:3.5.4.16]** | **0.019** | **0.814** |
| K01782 | 3-hydroxyacyl-CoA dehydrogenase / enoyl-CoA hydratase / 3-hydroxybutyryl-CoA epimerase [EC:1.1.1.35 4.2.1.17 5.1.2.3] | 0.005 | 0.898 |
| **K01825** | **3-hydroxyacyl-CoA dehydrogenase / enoyl-CoA hydratase / 3-hydroxybutyryl-CoA epimerase / enoyl-CoA isomerase [EC:1.1.1.35 4.2.1.17 5.1.2.3 5.3.3.8]** | **0.013** | **0.862** |
| K02274 | cytochrome c oxidase subunit I [EC:1.9.3.1] | 0.001 | 0.966 |
| K02275 | cytochrome c oxidase subunit II [EC:1.9.3.1] | 0.002 | 0.940 |
| **K02297** | **cytochrome o ubiquinol oxidase subunit II [EC:1.10.3.-]** | **0.014** | **0.852** |
| **K02298** | **cytochrome o ubiquinol oxidase subunit I [EC:1.10.3.-]** | **0.011** | **0.874** |
| **K02299** | **cytochrome o ubiquinol oxidase subunit III [EC:1.10.3.-]** | **0.012** | **0.865** |
| **K02300** | **cytochrome o ubiquinol oxidase operon protein cyoD** | **0.016** | **0.852** |
| K02472 | UDP-N-acetyl-D-mannosaminuronic acid dehydrogenase [EC:1.1.1.-] | 0.012 | 0.844 |
| **K02474** | **UDP-N-acetyl-D-galactosamine dehydrogenase [EC:1.1.1.-]** | **0.017** | **0.835** |
| **K02495** | **oxygen-independent coproporphyrinogen III oxidase [EC:1.3.99.22]** | **0.013** | **0.858** |
| K03185 | 2-octaprenyl-6-methoxyphenol hydroxylase [EC:1.14.13.-] | 0.037 | 0.746 |
| **K03336** | **3D-(3,5/4)-trihydroxycyclohexane-1,2-dione hydrolase [EC:3.7.1.-]** | **0.002** | **0.949** |
| **K03379** | **cyclohexanone monooxygenase [EC:1.14.13.22]** | **0.037** | **0.746** |
| K03381 | catechol 1,2-dioxygenase [EC:1.13.11.1] | 0.018 | 0.814 |
| **K03782** | **catalase/peroxidase [EC:1.11.1.6 1.11.1.7]** | **0.014** | **0.860** |
| K03863 | vanillate monooxygenase [EC:1.14.13.82] | 0.022 | 0.797 |
| **K04118** | **pimeloyl-CoA dehydrogenase [EC:1.3.1.62]** | **0.023** | **0.803** |
| **K05358** | **quinate dehydrogenase (quinone) [EC:1.1.5.8];quinate dehydrogenase (pyrroloquinoline-quinone) [EC:1.1.99.25]** | **0.019** | **0.848** |
| **K06134** | **ubiquinone biosynthesis monooxygenase Coq7 [EC:1.14.13.-]** | **0.018** | **0.822** |
| **K06445** | **acyl-CoA dehydrogenase [EC:1.3.99.-]** | **0.011** | **0.847** |
| **K06447** | **succinylglutamic semialdehyde dehydrogenase [EC:1.2.1.71]** | **0.015** | **0.831** |
| **K07516** | **3-hydroxyacyl-CoA dehydrogenase [EC:1.1.1.35]** | **0.012** | **0.869** |
| K08685 | quinohemoprotein amine dehydrogenase [EC:1.4.98.1];amine dehydrogenase [EC:1.4.99.3] | 0.003 | 0.915 |
| **K09471** | **gamma-glutamylputrescine oxidase [EC:1.4.3.-]** | **0.010** | **0.868** |
| **K09472** | **gamma-glutamyl-gamma-aminobutyraldehyde dehydrogenase [EC:1.2.1.-]** | **0.002** | **0.936** |
| K10216 | 2-hydroxymuconate-semialdehyde hydrolase [EC:3.7.1.9] | 0.031 | 0.763 |
| K10805 | acyl-CoA thioesterase II [EC:3.1.2.-] | 0.026 | 0.780 |
| **K10806** | **acyl-CoA thioesterase YciA [EC:3.1.2.-]** | **0.023** | **0.831** |
| K10946 | ammonia monooxygenase subunit C [EC:1.13.12.-] | 0.026 | 0.785 |
| K11755 | phosphoribosyl-ATP pyrophosphohydrolase / phosphoribosyl-AMP cyclohydrolase [EC:3.6.1.31 3.5.4.19] | 0.014 | 0.831 |
| K12251 | N-carbamoylputrescine amidase [EC:3.5.1.53] | 0.014 | 0.836 |
| **K12254** | **4-guanidinobutyraldehyde dehydrogenase / NAD-dependent aldehyde dehydrogenase [EC:1.2.1.54 1.2.1.-]** | **0.006** | **0.901** |
| **K12256** | **putrescine aminotransferase [EC:2.6.1.-]** | **0.005** | **0.915** |
| **K12524** | **bifunctional aspartokinase/homoserine dehydrogenase 1 [EC:2.7.2.4 1.1.1.3];bifunctional aspartokinase / homoserine dehydrogenase 1 [EC:2.7.2.4 1.1.1.3]** | **0.004** | **0.907** |
| K12979 | beta-hydroxylase [EC:1.14.11.-] | 0.017 | 0.818 |
| K13020 | UDP-D-GlcNAcA oxidase [EC:1.1.1.-] | 0.019 | 0.809 |
| **K13378** | **NADH dehydrogenase I subunit C/D [EC:1.6.5.3];NADH-quinone oxidoreductase subunit C/D [EC:1.6.5.3]** | **0.026** | **0.802** |
| **K13821** | **proline dehydrogenase / delta 1-pyrroline-5-carboxylate dehydrogenase [EC:1.5.99.8 1.5.1.12]** | **0.009** | **0.877** |
| **K13953** | **alcohol dehydrogenase, propanol-preferring [EC:1.1.1.1]** | **0.009** | **0.873** |
| **K13954** | **alcohol dehydrogenase [EC:1.1.1.1]** | **0.009** | **0.880** |
| **K14260** | **alanine-synthesizing transaminase [EC:2.6.1.66 2.6.1.2]** | **0.031** | **0.768** |
| K14268 | 5-aminovalerate aminotransferase [EC:2.6.1.48] | 0.003 | 0.915 |
| K14519 | NADP-dependent aldehyde dehydrogenase [EC:1.2.1.4] | 0.001 | 0.949 |
| **EE2** | | | |
| **KEGG Orthology number** | **Description** | ***P*-value** | **Spearman’s ρ** |
| K00001 | alcohol dehydrogenase [EC:1.1.1.1] | 0.037 | 0.458 |
| **K00003** | **homoserine dehydrogenase [EC:1.1.1.3]** | **0.028** | **0.530** |
| K00005 | glycerol dehydrogenase [EC:1.1.1.6] | 0.001 | 0.763 |
| **K00010** | **myo-inositol 2-dehydrogenase [EC:1.1.1.18]** | **0.022** | **0.642** |
| **K00012** | **UDPglucose 6-dehydrogenase [EC:1.1.1.22]** | **0.026** | **0.657** |
| **K00013** | **histidinol dehydrogenase [EC:1.1.1.23]** | **0.021** | **0.648** |
| **K00014** | **shikimate dehydrogenase [EC:1.1.1.25];shikimate 5-dehydrogenase [EC:1.1.1.25]** | **0.006** | **0.576** |
| K00016 | L-lactate dehydrogenase [EC:1.1.1.27] | 0.044 | 0.373 |
| K00018 | glycerate dehydrogenase [EC:1.1.1.29] | 0.003 | 0.627 |
| K00019 | 3-hydroxybutyrate dehydrogenase [EC:1.1.1.30] | 0.005 | 0.644 |
| **K00020** | **3-hydroxyisobutyrate dehydrogenase [EC:1.1.1.31]** | **0.015** | **0.619** |
| **K00024** | **malate dehydrogenase [EC:1.1.1.37]** | **0.009** | **0.696** |
| **K00027** | **malate dehydrogenase (oxaloacetate-decarboxylating) [EC:1.1.1.38]** | **0.027** | **0.492** |
| **K00029** | **malate dehydrogenase (oxaloacetate-decarboxylating)(NADP+) [EC:1.1.1.40]** | **0.013** | **0.648** |
| **K00031** | **isocitrate dehydrogenase [EC:1.1.1.42]** | **0.014** | **0.679** |
| K00052 | 3-isopropylmalate dehydrogenase [EC:1.1.1.85] | 0.014 | 0.424 |
| K00055 | aryl-alcohol dehydrogenase [EC:1.1.1.90] | 0.018 | 0.661 |
| K00057 | glycerol-3-phosphate dehydrogenase (NAD(P)+) [EC:1.1.1.94] | 0.012 | 0.581 |
| **K00058** | **D-3-phosphoglycerate dehydrogenase [EC:1.1.1.95]** | **0.018** | **0.665** |
| **K00074** | **3-hydroxybutyryl-CoA dehydrogenase [EC:1.1.1.157]** | **0.029** | **0.417** |
| K00075 | UDP-N-acetylmuramate dehydrogenase [EC:1.1.1.158] | 0.026 | 0.695 |
| **K00088** | **IMP dehydrogenase [EC:1.1.1.205]** | **0.012** | **0.654** |
| K00102 | D-lactate dehydrogenase (cytochrome) [EC:1.1.2.4] | 0.003 | 0.797 |
| **K00111** | **glycerol-3-phosphate dehydrogenase [EC:1.1.5.3]** | **0.013** | **0.557** |
| **K00116** | **malate dehydrogenase (quinone) [EC:1.1.5.4]** | **0.012** | **0.574** |
| **K00117** | **quinoprotein glucose dehydrogenase [EC:1.1.5.2]** | **0.022** | **0.455** |
| **K00121** | **S-(hydroxymethyl)glutathione dehydrogenase / alcohol dehydrogenase [EC:1.1.1.284 1.1.1.1]** | **0.013** | **0.616** |
| **K00123** | **formate dehydrogenase, alpha subunit [EC:1.2.1.2]** | **0.009** | **0.693** |
| **K00124** | **formate dehydrogenase, beta subunit [EC:1.2.1.2]** | **0.006** | **0.771** |
| **K00127** | **formate dehydrogenase, gamma subunit [EC:1.2.1.2]** | **0.004** | **0.694** |
| **K00128** | **aldehyde dehydrogenase (NAD+) [EC:1.2.1.3]** | **0.009** | **0.550** |
| K00130 | betaine-aldehyde dehydrogenase [EC:1.2.1.8] | 0.005 | 0.644 |
| **K00133** | **aspartate-semialdehyde dehydrogenase [EC:1.2.1.11]** | **0.007** | **0.655** |
| **K00134** | **glyceraldehyde 3-phosphate dehydrogenase [EC:1.2.1.12]** | **0.014** | **0.642** |
| **K00135** | **succinate-semialdehyde dehydrogenase (NADP+) [EC:1.2.1.16]** | **0.017** | **0.542** |
| **K00140** | **malonate-semialdehyde dehydrogenase (acetylating) / methylmalonate-semialdehyde dehydrogenase [EC:1.2.1.18 1.2.1.27];methylmalonate-semialdehyde dehydrogenase [EC:1.2.1.27]** | **0.013** | **0.629** |
| K00146 | phenylacetaldehyde dehydrogenase [EC:1.2.1.39] | 0.006 | 0.661 |
| **K00147** | **glutamate-5-semialdehyde dehydrogenase [EC:1.2.1.41]** | **0.007** | **0.695** |
| K00156 | pyruvate dehydrogenase (quinone) [EC:1.2.5.1];pyruvate dehydrogenase (cytochrome) [EC:1.2.2.2] | 0.022 | 0.373 |
| **K00161** | **pyruvate dehydrogenase E1 component subunit alpha [EC:1.2.4.1]** | **0.012** | **0.499** |
| K00162 | pyruvate dehydrogenase E1 component subunit beta [EC:1.2.4.1] | 0.031 | 0.271 |
| **K00163** | **pyruvate dehydrogenase E1 component [EC:1.2.4.1]** | **0.009** | **0.669** |
| **K00164** | **2-oxoglutarate dehydrogenase E1 component [EC:1.2.4.2]** | **0.009** | **0.648** |
| **K00166** | **2-oxoisovalerate dehydrogenase E1 component, alpha subunit [EC:1.2.4.4]** | **0.006** | **0.746** |
| K00228 | coproporphyrinogen III oxidase [EC:1.3.3.3] | 0.003 | 0.797 |
| **K00239** | **succinate dehydrogenase flavoprotein subunit [EC:1.3.99.1]** | **0.011** | **0.627** |
| **K00240** | **succinate dehydrogenase iron-sulfur protein [EC:1.3.99.1];succinate dehydrogenase iron-sulfur subunit [EC:1.3.99.1]** | **0.014** | **0.632** |
| **K00241** | **succinate dehydrogenase cytochrome b556 subunit;succinate dehydrogenase cytochrome b-556 subunit [EC:1.3.99.1];succinate dehydrogenase cytochrome b-556 subunit** | **0.015** | **0.581** |
| K00242 | succinate dehydrogenase hydrophobic membrane anchor protein [EC:1.3.99.1];succinate dehydrogenase hydrophobic membrane anchor protein;succinate dehydrogenase membrane anchor subunit | 0.044 | 0.695 |
| K00249 | acyl-CoA dehydrogenase [EC:1.3.99.3] | 0.017 | 0.592 |
| **K00252** | **glutaryl-CoA dehydrogenase [EC:1.3.99.7]** | **0.010** | **0.579** |
| K00253 | isovaleryl-CoA dehydrogenase [EC:1.3.99.10] | 0.003 | 0.712 |
| **K00254** | **dihydroorotate dehydrogenase [EC:1.3.5.2]** | **0.023** | **0.619** |
| K00255 | long-chain-acyl-CoA dehydrogenase [EC:1.3.99.13] | 0.006 | 0.763 |
| **K00259** | **alanine dehydrogenase [EC:1.4.1.1]** | **0.010** | **0.689** |
| **K00261** | **glutamate dehydrogenase (NAD(P)+) [EC:1.4.1.3]** | **0.008** | **0.696** |
| **K00262** | **glutamate dehydrogenase (NADP+) [EC:1.4.1.4]** | **0.012** | **0.603** |
| **K00263** | **leucine dehydrogenase [EC:1.4.1.9]** | **0.004** | **0.687** |
| K00274 | monoamine oxidase [EC:1.4.3.4] | 0.001 | 0.763 |
| **K00281** | **glycine dehydrogenase [EC:1.4.4.2]** | **0.009** | **0.575** |
| **K00285** | **D-amino-acid dehydrogenase [EC:1.4.99.1]** | **0.013** | **0.614** |
| **K00294** | **1-pyrroline-5-carboxylate dehydrogenase [EC:1.5.1.12]** | **0.028** | **0.390** |
| **K00318** | **proline dehydrogenase [EC:1.5.99.8]** | **0.020** | **0.424** |
| **K00330** | **NADH-quinone oxidoreductase subunit A [EC:1.6.5.3];NADH dehydrogenase I subunit A [EC:1.6.5.3]** | **0.009** | **0.641** |
| **K00331** | **NADH dehydrogenase I subunit B [EC:1.6.5.3];NADH-quinone oxidoreductase subunit B [EC:1.6.5.3]** | **0.014** | **0.658** |
| **K00332** | **NADH-quinone oxidoreductase subunit C [EC:1.6.5.3];NADH dehydrogenase I subunit C [EC:1.6.5.3]** | **0.004** | **0.759** |
| **K00333** | **NADH dehydrogenase I subunit D [EC:1.6.5.3];NADH-quinone oxidoreductase subunit D [EC:1.6.5.3]** | **0.006** | **0.665** |
| **K00334** | **NADH-quinone oxidoreductase subunit E [EC:1.6.5.3];NADH dehydrogenase I subunit E [EC:1.6.5.3]** | **0.012** | **0.578** |
| **K00335** | **NADH dehydrogenase I subunit F [EC:1.6.5.3];NADH-quinone oxidoreductase subunit F [EC:1.6.5.3]** | **0.009** | **0.721** |
| **K00336** | **NADH-quinone oxidoreductase subunit G [EC:1.6.5.3];NADH dehydrogenase I subunit G [EC:1.6.5.3]** | **0.012** | **0.642** |
| **K00337** | **NADH dehydrogenase I subunit H [EC:1.6.5.3];NADH-quinone oxidoreductase subunit H [EC:1.6.5.3]** | **0.016** | **0.648** |
| **K00338** | **NADH-quinone oxidoreductase subunit I [EC:1.6.5.3];NADH dehydrogenase I subunit I [EC:1.6.5.3]** | **0.016** | **0.588** |
| **K00339** | **NADH dehydrogenase I subunit J [EC:1.6.5.3];NADH-quinone oxidoreductase subunit J [EC:1.6.5.3]** | **0.008** | **0.683** |
| **K00340** | **NADH-quinone oxidoreductase subunit K [EC:1.6.5.3];NADH dehydrogenase I subunit K [EC:1.6.5.3]** | **0.022** | **0.619** |
| **K00341** | **NADH dehydrogenase I subunit L [EC:1.6.5.3];NADH-quinone oxidoreductase subunit L [EC:1.6.5.3]** | **0.010** | **0.635** |
| **K00342** | **NADH-quinone oxidoreductase subunit M [EC:1.6.5.3];NADH dehydrogenase I subunit M [EC:1.6.5.3]** | **0.017** | **0.640** |
| **K00343** | **NADH dehydrogenase I subunit N [EC:1.6.5.3];NADH-quinone oxidoreductase subunit N [EC:1.6.5.3]** | **0.017** | **0.658** |
| **K00382** | **dihydrolipoamide dehydrogenase [EC:1.8.1.4]** | **0.013** | **0.645** |
| **K00404** | **cb-type cytochrome c oxidase subunit I [EC:1.9.3.1];cytochrome c oxidase cb-type subunit I [EC:1.9.3.1]** | **0.008** | **0.681** |
| **K00405** | **cb-type cytochrome c oxidase subunit II [EC:1.9.3.1];cytochrome c oxidase cb-type subunit II** | **0.005** | **0.743** |
| **K00406** | **cb-type cytochrome c oxidase subunit III [EC:1.9.3.1];cytochrome c oxidase cb-type subunit III** | **0.005** | **0.669** |
| **K00425** | **cytochrome d ubiquinol oxidase subunit I [EC:1.10.3.-];cytochrome bd-I oxidase subunit I [EC:1.10.3.-]** | **0.013** | **0.613** |
| **K00426** | **cytochrome d ubiquinol oxidase subunit II [EC:1.10.3.-];cytochrome bd-I oxidase subunit II [EC:1.10.3.-]** | **0.016** | **0.642** |
| **K00432** | **glutathione peroxidase [EC:1.11.1.9]** | **0.020** | **0.490** |
| **K00457** | **4-hydroxyphenylpyruvate dioxygenase [EC:1.13.11.27]** | **0.013** | **0.604** |
| **K00459** | **nitronate monooxygenase [EC:1.13.12.16]** | **0.007** | **0.681** |
| **K00466** | **tryptophan 2-monooxygenase [EC:1.13.12.3]** | **0.003** | **0.627** |
| **K00499** | **choline monooxygenase [EC:1.14.15.7]** | **0.019** | **0.486** |
| **K00500** | **phenylalanine-4-hydroxylase [EC:1.14.16.1]** | **0.010** | **0.625** |
| **K00602** | **phosphoribosylaminoimidazolecarboxamide formyltransferase / IMP cyclohydrolase [EC:2.1.2.3 3.5.4.10]** | **0.016** | **0.672** |
| **K00627** | **pyruvate dehydrogenase E2 component (dihydrolipoamide acetyltransferase) [EC:2.3.1.12]** | **0.021** | **0.457** |
| **K00658** | **2-oxoglutarate dehydrogenase E2 component (dihydrolipoamide succinyltransferase) [EC:2.3.1.61]** | **0.014** | **0.610** |
| **K00812** | **aspartate aminotransferase [EC:2.6.1.1]** | **0.019** | **0.652** |
| **K00817** | **histidinol-phosphate aminotransferase [EC:2.6.1.9]** | **0.013** | **0.729** |
| **K00818** | **acetylornithine aminotransferase [EC:2.6.1.11]** | **0.006** | **0.650** |
| **K00820** | **glucosamine--fructose-6-phosphate aminotransferase (isomerizing) [EC:2.6.1.16]** | **0.024** | **0.395** |
| **K00821** | **acetylornithine/N-succinyldiaminopimelate aminotransferase [EC:2.6.1.11 2.6.1.17]** | **0.007** | **0.614** |
| **K00822** | **beta-alanine--pyruvate transaminase [EC:2.6.1.18]** | **0.005** | **0.746** |
| **K00823** | **4-aminobutyrate aminotransferase [EC:2.6.1.19]** | **0.005** | **0.688** |
| **K00826** | **branched-chain amino acid aminotransferase [EC:2.6.1.42]** | **0.014** | **0.564** |
| **K00831** | **phosphoserine aminotransferase [EC:2.6.1.52]** | **0.020** | **0.592** |
| **K00832** | **aromatic-amino-acid transaminase [EC:2.6.1.57]** | **0.029** | **0.415** |
| **K00836** | **diaminobutyrate-2-oxoglutarate transaminase [EC:2.6.1.76]** | **0.022** | **0.631** |
| K01066 | esterase / lipase [EC:3.1.1.-] | 0.044 | 0.373 |
| **K01067** | **acetyl-CoA hydrolase [EC:3.1.2.1]** | **0.023** | **0.502** |
| **K01139** | **guanosine-3',5'-bis(diphosphate) 3'-pyrophosphohydrolase [EC:3.1.7.2]** | **0.017** | **0.589** |
| **K01426** | **amidase [EC:3.5.1.4]** | **0.020** | **0.449** |
| K01434 | penicillin amidase [EC:3.5.1.11] | 0.026 | 0.305 |
| K01436 | aminoacylase [EC:3.5.1.14];amidohydrolase [EC:3.5.1.-] | 0.005 | 0.593 |
| **K01442** | **choloylglycine hydrolase [EC:3.5.1.24]** | **0.015** | **0.653** |
| **K01484** | **succinylarginine dihydrolase [EC:3.5.3.23]** | **0.013** | **0.610** |
| **K01491** | **methylenetetrahydrofolate dehydrogenase (NADP+) / methenyltetrahydrofolate cyclohydrolase [EC:1.5.1.5 3.5.4.9]** | **0.005** | **0.610** |
| **K01495** | **GTP cyclohydrolase I [EC:3.5.4.16]** | **0.019** | **0.720** |
| K01782 | 3-hydroxyacyl-CoA dehydrogenase / enoyl-CoA hydratase / 3-hydroxybutyryl-CoA epimerase [EC:1.1.1.35 4.2.1.17 5.1.2.3] | 0.005 | 0.814 |
| **K01825** | **3-hydroxyacyl-CoA dehydrogenase / enoyl-CoA hydratase / 3-hydroxybutyryl-CoA epimerase / enoyl-CoA isomerase [EC:1.1.1.35 4.2.1.17 5.1.2.3 5.3.3.8]** | **0.013** | **0.616** |
| K02274 | cytochrome c oxidase subunit I [EC:1.9.3.1] | 0.001 | 0.746 |
| K02275 | cytochrome c oxidase subunit II [EC:1.9.3.1] | 0.002 | 0.775 |
| **K02297** | **cytochrome o ubiquinol oxidase subunit II [EC:1.10.3.-]** | **0.014** | **0.647** |
| **K02298** | **cytochrome o ubiquinol oxidase subunit I [EC:1.10.3.-]** | **0.011** | **0.754** |
| **K02299** | **cytochrome o ubiquinol oxidase subunit III [EC:1.10.3.-]** | **0.012** | **0.716** |
| **K02300** | **cytochrome o ubiquinol oxidase operon protein cyoD** | **0.016** | **0.640** |
| K02472 | UDP-N-acetyl-D-mannosaminuronic acid dehydrogenase [EC:1.1.1.-] | 0.012 | 0.870 |
| **K02474** | **UDP-N-acetyl-D-galactosamine dehydrogenase [EC:1.1.1.-]** | **0.017** | **0.659** |
| **K02495** | **oxygen-independent coproporphyrinogen III oxidase [EC:1.3.99.22]** | **0.013** | **0.649** |
| K03185 | 2-octaprenyl-6-methoxyphenol hydroxylase [EC:1.14.13.-] | 0.037 | 0.288 |
| **K03336** | **3D-(3,5/4)-trihydroxycyclohexane-1,2-dione hydrolase [EC:3.7.1.-]** | **0.002** | **0.720** |
| **K03379** | **cyclohexanone monooxygenase [EC:1.14.13.22]** | **0.037** | **0.348** |
| K03381 | catechol 1,2-dioxygenase [EC:1.13.11.1] | 0.018 | 0.593 |
| **K03782** | **catalase/peroxidase [EC:1.11.1.6 1.11.1.7]** | **0.014** | **0.577** |
| K03863 | vanillate monooxygenase [EC:1.14.13.82] | 0.022 | 0.525 |
| K04118 | pimeloyl-CoA dehydrogenase [EC:1.3.1.62] | 0.023 | 0.812 |
| **K05358** | **quinate dehydrogenase (quinone) [EC:1.1.5.8];quinate dehydrogenase (pyrroloquinoline-quinone) [EC:1.1.99.25]** | **0.019** | **0.441** |
| **K06134** | **ubiquinone biosynthesis monooxygenase Coq7 [EC:1.14.13.-]** | **0.018** | **0.665** |
| **K06445** | **acyl-CoA dehydrogenase [EC:1.3.99.-]** | **0.011** | **0.801** |
| **K06447** | **succinylglutamic semialdehyde dehydrogenase [EC:1.2.1.71]** | **0.015** | **0.449** |
| **K07516** | **3-hydroxyacyl-CoA dehydrogenase [EC:1.1.1.35]** | **0.012** | **0.711** |
| K08685 | quinohemoprotein amine dehydrogenase [EC:1.4.98.1];amine dehydrogenase [EC:1.4.99.3] | 0.003 | 0.627 |
| **K09471** | **gamma-glutamylputrescine oxidase [EC:1.4.3.-]** | **0.010** | **0.540** |
| **K09472** | **gamma-glutamyl-gamma-aminobutyraldehyde dehydrogenase [EC:1.2.1.-]** | **0.002** | **0.692** |
| K10216 | 2-hydroxymuconate-semialdehyde hydrolase [EC:3.7.1.9] | 0.031 | 0.475 |
| K10805 | acyl-CoA thioesterase II [EC:3.1.2.-] | 0.026 | 0.627 |
| **K10806** | **acyl-CoA thioesterase YciA [EC:3.1.2.-]** | **0.023** | **0.551** |
| K10946 | ammonia monooxygenase subunit C [EC:1.13.12.-] | 0.026 | 0.694 |
| K11755 | phosphoribosyl-ATP pyrophosphohydrolase / phosphoribosyl-AMP cyclohydrolase [EC:3.6.1.31 3.5.4.19] | 0.014 | 0.407 |
| K12251 | N-carbamoylputrescine amidase [EC:3.5.1.53] | 0.014 | 0.574 |
| **K12254** | **4-guanidinobutyraldehyde dehydrogenase / NAD-dependent aldehyde dehydrogenase [EC:1.2.1.54 1.2.1.-]** | **0.006** | **0.618** |
| **K12256** | **putrescine aminotransferase [EC:2.6.1.-]** | **0.005** | **0.667** |
| **K12524** | **bifunctional aspartokinase/homoserine dehydrogenase 1 [EC:2.7.2.4 1.1.1.3];bifunctional aspartokinase / homoserine dehydrogenase 1 [EC:2.7.2.4 1.1.1.3]** | **0.004** | **0.729** |
| K12979 | beta-hydroxylase [EC:1.14.11.-] | 0.017 | 0.592 |
| K13020 | UDP-D-GlcNAcA oxidase [EC:1.1.1.-] | 0.019 | 0.818 |
| **K13378** | **NADH dehydrogenase I subunit C/D [EC:1.6.5.3];NADH-quinone oxidoreductase subunit C/D [EC:1.6.5.3]** | **0.026** | **0.683** |
| **K13821** | **proline dehydrogenase / delta 1-pyrroline-5-carboxylate dehydrogenase [EC:1.5.99.8 1.5.1.12]** | **0.009** | **0.677** |
| **K13953** | **alcohol dehydrogenase, propanol-preferring [EC:1.1.1.1]** | **0.009** | **0.639** |
| **K13954** | **alcohol dehydrogenase [EC:1.1.1.1]** | **0.009** | **0.636** |
| **K14260** | **alanine-synthesizing transaminase [EC:2.6.1.66 2.6.1.2]** | **0.031** | **0.605** |
| K14268 | 5-aminovalerate aminotransferase [EC:2.6.1.48] | 0.003 | 0.712 |
| K14519 | NADP-dependent aldehyde dehydrogenase [EC:1.2.1.4] | 0.001 | 0.678 |
| **Trimethoprim** | | | |
| **KEGG Orthology number** | **Description** | ***P*-value** | **Spearman’s ρ** |
| K00020 | 3-hydroxyisobutyrate dehydrogenase [EC:1.1.1.31] | 0.027 | 0.953 |
| K00031 | isocitrate dehydrogenase [EC:1.1.1.42] | 0.027 | 0.953 |
| K00333 | NADH dehydrogenase I subunit D [EC:1.6.5.3];NADH-quinone oxidoreductase subunit D [EC:1.6.5.3] | 0.027 | 0.953 |
| K00339 | NADH dehydrogenase I subunit J [EC:1.6.5.3];NADH-quinone oxidoreductase subunit J [EC:1.6.5.3] | 0.027 | 0.953 |
| K06445 | acyl-CoA dehydrogenase [EC:1.3.99.-] | 0.027 | 0.953 |
| **Venlafaxine** | | | |
| **KEGG Orthology number** | **Description** | ***P*-value** | **Spearman’s ρ** |
| K00001 | alcohol dehydrogenase [EC:1.1.1.1] | 0.022 | 0.797 |
| **K00003** | **homoserine dehydrogenase [EC:1.1.1.3]** | **0.018** | **0.831** |
| K00005 | glycerol dehydrogenase [EC:1.1.1.6] | 0.001 | 0.966 |
| **K00010** | **myo-inositol 2-dehydrogenase [EC:1.1.1.18]** | **0.020** | **0.815** |
| **K00012** | **UDPglucose 6-dehydrogenase [EC:1.1.1.22]** | **0.014** | **0.834** |
| **K00013** | **histidinol dehydrogenase [EC:1.1.1.23]** | **0.016** | **0.839** |
| **K00014** | **shikimate dehydrogenase [EC:1.1.1.25];shikimate 5-dehydrogenase [EC:1.1.1.25]** | **0.010** | **0.865** |
| K00018 | glycerate dehydrogenase [EC:1.1.1.29] | 0.005 | 0.898 |
| K00019 | 3-hydroxybutyrate dehydrogenase [EC:1.1.1.30] | 0.008 | 0.865 |
| **K00020** | **3-hydroxyisobutyrate dehydrogenase [EC:1.1.1.31]** | **0.019** | **0.841** |
| **K00024** | **malate dehydrogenase [EC:1.1.1.37]** | **0.010** | **0.876** |
| **K00027** | **malate dehydrogenase (oxaloacetate-decarboxylating) [EC:1.1.1.38]** | **0.020** | **0.808** |
| **K00029** | **malate dehydrogenase (oxaloacetate-decarboxylating)(NADP+) [EC:1.1.1.40]** | **0.012** | **0.861** |
| **K00031** | **isocitrate dehydrogenase [EC:1.1.1.42]** | **0.013** | **0.854** |
| **K00052** | **3-isopropylmalate dehydrogenase [EC:1.1.1.85]** | **0.031** | **0.763** |
| K00055 | aryl-alcohol dehydrogenase [EC:1.1.1.90] | 0.026 | 0.780 |
| **K00057** | **glycerol-3-phosphate dehydrogenase (NAD(P)+) [EC:1.1.1.94]** | **0.027** | **0.793** |
| **K00058** | **D-3-phosphoglycerate dehydrogenase [EC:1.1.1.95]** | **0.021** | **0.820** |
| **K00074** | **3-hydroxybutyryl-CoA dehydrogenase [EC:1.1.1.157]** | **0.022** | **0.797** |
| K00075 | UDP-N-acetylmuramate dehydrogenase [EC:1.1.1.158] | 0.018 | 0.814 |
| **K00088** | **IMP dehydrogenase [EC:1.1.1.205]** | **0.009** | **0.874** |
| **K00097** | **4-hydroxythreonine-4-phosphate dehydrogenase [EC:1.1.1.262]** | **0.044** | **0.729** |
| K00102 | D-lactate dehydrogenase (cytochrome) [EC:1.1.2.4] | 0.002 | 0.949 |
| **K00111** | **glycerol-3-phosphate dehydrogenase [EC:1.1.5.3]** | **0.008** | **0.890** |
| **K00116** | **malate dehydrogenase (quinone) [EC:1.1.5.4]** | **0.011** | **0.876** |
| **K00117** | **quinoprotein glucose dehydrogenase [EC:1.1.5.2]** | **0.025** | **0.785** |
| **K00121** | **S-(hydroxymethyl)glutathione dehydrogenase / alcohol dehydrogenase [EC:1.1.1.284 1.1.1.1]** | **0.013** | **0.844** |
| **K00123** | **formate dehydrogenase, alpha subunit [EC:1.2.1.2]** | **0.009** | **0.889** |
| **K00124** | **formate dehydrogenase, beta subunit [EC:1.2.1.2]** | **0.003** | **0.932** |
| **K00127** | **formate dehydrogenase, gamma subunit [EC:1.2.1.2]** | **0.007** | **0.883** |
| **K00128** | **aldehyde dehydrogenase (NAD+) [EC:1.2.1.3]** | **0.015** | **0.832** |
| K00130 | betaine-aldehyde dehydrogenase [EC:1.2.1.8] | 0.003 | 0.915 |
| **K00133** | **aspartate-semialdehyde dehydrogenase [EC:1.2.1.11]** | **0.007** | **0.892** |
| **K00134** | **glyceraldehyde 3-phosphate dehydrogenase [EC:1.2.1.12]** | **0.014** | **0.848** |
| **K00135** | **succinate-semialdehyde dehydrogenase (NADP+) [EC:1.2.1.16]** | **0.012** | **0.862** |
| **K00140** | **malonate-semialdehyde dehydrogenase (acetylating) / methylmalonate-semialdehyde dehydrogenase [EC:1.2.1.18 1.2.1.27];methylmalonate-semialdehyde dehydrogenase [EC:1.2.1.27]** | **0.016** | **0.840** |
| K00146 | phenylacetaldehyde dehydrogenase [EC:1.2.1.39] | 0.008 | 0.865 |
| **K00147** | **glutamate-5-semialdehyde dehydrogenase [EC:1.2.1.41]** | **0.004** | **0.912** |
| K00156 | pyruvate dehydrogenase (quinone) [EC:1.2.5.1];pyruvate dehydrogenase (cytochrome) [EC:1.2.2.2] | 0.031 | 0.763 |
| **K00161** | **pyruvate dehydrogenase E1 component subunit alpha [EC:1.2.4.1]** | **0.018** | **0.837** |
| K00162 | pyruvate dehydrogenase E1 component subunit beta [EC:1.2.4.1] | 0.044 | 0.729 |
| **K00163** | **pyruvate dehydrogenase E1 component [EC:1.2.4.1]** | **0.013** | **0.864** |
| **K00164** | **2-oxoglutarate dehydrogenase E1 component [EC:1.2.4.2]** | **0.012** | **0.865** |
| **K00166** | **2-oxoisovalerate dehydrogenase E1 component, alpha subunit [EC:1.2.4.4]** | **0.006** | **0.890** |
| K00228 | coproporphyrinogen III oxidase [EC:1.3.3.3] | 0.003 | 0.932 |
| **K00239** | **succinate dehydrogenase flavoprotein subunit [EC:1.3.99.1]** | **0.010** | **0.874** |
| **K00240** | **succinate dehydrogenase iron-sulfur protein [EC:1.3.99.1];succinate dehydrogenase iron-sulfur subunit [EC:1.3.99.1]** | **0.021** | **0.819** |
| **K00241** | **succinate dehydrogenase cytochrome b556 subunit;succinate dehydrogenase cytochrome b-556 subunit [EC:1.3.99.1];succinate dehydrogenase cytochrome b-556 subunit** | **0.015** | **0.845** |
| K00242 | succinate dehydrogenase hydrophobic membrane anchor protein [EC:1.3.99.1];succinate dehydrogenase hydrophobic membrane anchor protein;succinate dehydrogenase membrane anchor subunit | 0.031 | 0.763 |
| K00249 | acyl-CoA dehydrogenase [EC:1.3.99.3] | 0.026 | 0.783 |
| **K00252** | **glutaryl-CoA dehydrogenase [EC:1.3.99.7]** | **0.014** | **0.845** |
| K00253 | isovaleryl-CoA dehydrogenase [EC:1.3.99.10] | 0.005 | 0.898 |
| **K00254** | **dihydroorotate dehydrogenase [EC:1.3.5.2]** | **0.028** | **0.791** |
| K00255 | long-chain-acyl-CoA dehydrogenase [EC:1.3.99.13] | 0.003 | 0.932 |
| **K00259** | **alanine dehydrogenase [EC:1.4.1.1]** | **0.010** | **0.878** |
| **K00261** | **glutamate dehydrogenase (NAD(P)+) [EC:1.4.1.3]** | **0.009** | **0.869** |
| **K00262** | **glutamate dehydrogenase (NADP+) [EC:1.4.1.4]** | **0.020** | **0.811** |
| **K00263** | **leucine dehydrogenase [EC:1.4.1.9]** | **0.008** | **0.873** |
| K00274 | monoamine oxidase [EC:1.4.3.4] | 0.001 | 0.966 |
| **K00281** | **glycine dehydrogenase [EC:1.4.4.2]** | **0.012** | **0.865** |
| **K00285** | **D-amino-acid dehydrogenase [EC:1.4.99.1]** | **0.015** | **0.856** |
| **K00294** | **1-pyrroline-5-carboxylate dehydrogenase [EC:1.5.1.12]** | **0.003** | **0.922** |
| **K00318** | **proline dehydrogenase [EC:1.5.99.8]** | **0.027** | **0.788** |
| **K00330** | **NADH-quinone oxidoreductase subunit A [EC:1.6.5.3];NADH dehydrogenase I subunit A [EC:1.6.5.3]** | **0.009** | **0.874** |
| **K00331** | **NADH dehydrogenase I subunit B [EC:1.6.5.3];NADH-quinone oxidoreductase subunit B [EC:1.6.5.3]** | **0.016** | **0.835** |
| **K00332** | **NADH-quinone oxidoreductase subunit C [EC:1.6.5.3];NADH dehydrogenase I subunit C [EC:1.6.5.3]** | **0.005** | **0.902** |
| **K00333** | **NADH dehydrogenase I subunit D [EC:1.6.5.3];NADH-quinone oxidoreductase subunit D [EC:1.6.5.3]** | **0.010** | **0.885** |
| **K00334** | **NADH-quinone oxidoreductase subunit E [EC:1.6.5.3];NADH dehydrogenase I subunit E [EC:1.6.5.3]** | **0.018** | **0.838** |
| **K00335** | **NADH dehydrogenase I subunit F [EC:1.6.5.3];NADH-quinone oxidoreductase subunit F [EC:1.6.5.3]** | **0.006** | **0.893** |
| **K00336** | **NADH-quinone oxidoreductase subunit G [EC:1.6.5.3];NADH dehydrogenase I subunit G [EC:1.6.5.3]** | **0.012** | **0.862** |
| **K00337** | **NADH dehydrogenase I subunit H [EC:1.6.5.3];NADH-quinone oxidoreductase subunit H [EC:1.6.5.3]** | **0.019** | **0.826** |
| **K00338** | **NADH-quinone oxidoreductase subunit I [EC:1.6.5.3];NADH dehydrogenase I subunit I [EC:1.6.5.3]** | **0.016** | **0.866** |
| **K00339** | **NADH dehydrogenase I subunit J [EC:1.6.5.3];NADH-quinone oxidoreductase subunit J [EC:1.6.5.3]** | **0.009** | **0.878** |
| **K00340** | **NADH-quinone oxidoreductase subunit K [EC:1.6.5.3];NADH dehydrogenase I subunit K [EC:1.6.5.3]** | **0.022** | **0.797** |
| **K00341** | **NADH dehydrogenase I subunit L [EC:1.6.5.3];NADH-quinone oxidoreductase subunit L [EC:1.6.5.3]** | **0.015** | **0.847** |
| **K00342** | **NADH-quinone oxidoreductase subunit M [EC:1.6.5.3];NADH dehydrogenase I subunit M [EC:1.6.5.3]** | **0.019** | **0.825** |
| **K00343** | **NADH dehydrogenase I subunit N [EC:1.6.5.3];NADH-quinone oxidoreductase subunit N [EC:1.6.5.3]** | **0.017** | **0.830** |
| **K00382** | **dihydrolipoamide dehydrogenase [EC:1.8.1.4]** | **0.012** | **0.867** |
| **K00404** | **cb-type cytochrome c oxidase subunit I [EC:1.9.3.1];cytochrome c oxidase cb-type subunit I [EC:1.9.3.1]** | **0.008** | **0.882** |
| **K00405** | **cb-type cytochrome c oxidase subunit II [EC:1.9.3.1];cytochrome c oxidase cb-type subunit II** | **0.005** | **0.904** |
| **K00406** | **cb-type cytochrome c oxidase subunit III [EC:1.9.3.1];cytochrome c oxidase cb-type subunit III** | **0.007** | **0.898** |
| **K00425** | **cytochrome d ubiquinol oxidase subunit I [EC:1.10.3.-];cytochrome bd-I oxidase subunit I [EC:1.10.3.-]** | **0.016** | **0.843** |
| **K00426** | **cytochrome d ubiquinol oxidase subunit II [EC:1.10.3.-];cytochrome bd-I oxidase subunit II [EC:1.10.3.-]** | **0.016** | **0.843** |
| **K00432** | **glutathione peroxidase [EC:1.11.1.9]** | **0.016** | **0.836** |
| **K00457** | **4-hydroxyphenylpyruvate dioxygenase [EC:1.13.11.27]** | **0.010** | **0.875** |
| **K00459** | **nitronate monooxygenase [EC:1.13.12.16]** | **0.011** | **0.873** |
| K00466 | tryptophan 2-monooxygenase [EC:1.13.12.3] | 0.006 | 0.881 |
| **K00499** | **choline monooxygenase [EC:1.14.15.7]** | **0.008** | **0.898** |
| **K00500** | **phenylalanine-4-hydroxylase [EC:1.14.16.1]** | **0.009** | **0.882** |
| **K00602** | **phosphoribosylaminoimidazolecarboxamide formyltransferase / IMP cyclohydrolase [EC:2.1.2.3 3.5.4.10]** | **0.014** | **0.851** |
| **K00627** | **pyruvate dehydrogenase E2 component (dihydrolipoamide acetyltransferase) [EC:2.3.1.12]** | **0.026** | **0.803** |
| **K00658** | **2-oxoglutarate dehydrogenase E2 component (dihydrolipoamide succinyltransferase) [EC:2.3.1.61]** | **0.011** | **0.864** |
| **K00812** | **aspartate aminotransferase [EC:2.6.1.1]** | **0.018** | **0.834** |
| **K00817** | **histidinol-phosphate aminotransferase [EC:2.6.1.9]** | **0.014** | **0.856** |
| **K00818** | **acetylornithine aminotransferase [EC:2.6.1.11]** | **0.009** | **0.887** |
| **K00820** | **glucosamine--fructose-6-phosphate aminotransferase (isomerizing) [EC:2.6.1.16]** | **0.023** | **0.802** |
| **K00821** | **acetylornithine/N-succinyldiaminopimelate aminotransferase [EC:2.6.1.11 2.6.1.17]** | **0.012** | **0.851** |
| **K00822** | **beta-alanine--pyruvate transaminase [EC:2.6.1.18]** | **0.004** | **0.924** |
| **K00823** | **4-aminobutyrate aminotransferase [EC:2.6.1.19]** | **0.008** | **0.886** |
| **K00826** | **branched-chain amino acid aminotransferase [EC:2.6.1.42]** | **0.018** | **0.828** |
| **K00831** | **phosphoserine aminotransferase [EC:2.6.1.52]** | **0.018** | **0.820** |
| K00832 | aromatic-amino-acid transaminase [EC:2.6.1.57] | 0.044 | 0.729 |
| **K00836** | **diaminobutyrate-2-oxoglutarate transaminase [EC:2.6.1.76]** | **0.023** | **0.806** |
| K01066 | esterase / lipase [EC:3.1.1.-] | 0.031 | 0.763 |
| **K01067** | **acetyl-CoA hydrolase [EC:3.1.2.1]** | **0.029** | **0.779** |
| K01126 | glycerophosphoryl diester phosphodiesterase [EC:3.1.4.46] | 0.037 | 0.746 |
| **K01139** | **guanosine-3',5'-bis(diphosphate) 3'-pyrophosphohydrolase [EC:3.1.7.2]** | **0.021** | **0.814** |
| K01426 | amidase [EC:3.5.1.4] | 0.022 | 0.797 |
| K01434 | penicillin amidase [EC:3.5.1.11] | 0.031 | 0.763 |
| K01436 | aminoacylase [EC:3.5.1.14];amidohydrolase [EC:3.5.1.-] | 0.006 | 0.881 |
| **K01442** | **choloylglycine hydrolase [EC:3.5.1.24]** | **0.015** | **0.881** |
| **K01484** | **succinylarginine dihydrolase [EC:3.5.3.23]** | **0.009** | **0.881** |
| **K01491** | **methylenetetrahydrofolate dehydrogenase (NADP+) / methenyltetrahydrofolate cyclohydrolase [EC:1.5.1.5 3.5.4.9]** | **0.013** | **0.848** |
| **K01495** | **GTP cyclohydrolase I [EC:3.5.4.16]** | **0.008** | **0.873** |
| K01782 | 3-hydroxyacyl-CoA dehydrogenase / enoyl-CoA hydratase / 3-hydroxybutyryl-CoA epimerase [EC:1.1.1.35 4.2.1.17 5.1.2.3] | 0.003 | 0.915 |
| **K01825** | **3-hydroxyacyl-CoA dehydrogenase / enoyl-CoA hydratase / 3-hydroxybutyryl-CoA epimerase / enoyl-CoA isomerase [EC:1.1.1.35 4.2.1.17 5.1.2.3 5.3.3.8]** | **0.013** | **0.865** |
| K02274 | cytochrome c oxidase subunit I [EC:1.9.3.1] | 0.003 | 0.932 |
| K02275 | cytochrome c oxidase subunit II [EC:1.9.3.1] | 0.004 | 0.905 |
| **K02297** | **cytochrome o ubiquinol oxidase subunit II [EC:1.10.3.-]** | **0.010** | **0.871** |
| **K02298** | **cytochrome o ubiquinol oxidase subunit I [EC:1.10.3.-]** | **0.008** | **0.889** |
| **K02299** | **cytochrome o ubiquinol oxidase subunit III [EC:1.10.3.-]** | **0.007** | **0.894** |
| **K02300** | **cytochrome o ubiquinol oxidase operon protein cyoD** | **0.010** | **0.873** |
| K02302 | uroporphyrin-III C-methyltransferase / precorrin-2 dehydrogenase / sirohydrochlorin ferrochelatase [EC:2.1.1.107 1.3.1.76 4.99.1.4] | 0.031 | 0.763 |
| **K02472** | **UDP-N-acetyl-D-mannosaminuronic acid dehydrogenase [EC:1.1.1.-]** | **0.026** | **0.795** |
| **K02474** | **UDP-N-acetyl-D-galactosamine dehydrogenase [EC:1.1.1.-]** | **0.018** | **0.830** |
| **K02495** | **oxygen-independent coproporphyrinogen III oxidase [EC:1.3.99.22]** | **0.012** | **0.868** |
| **K03336** | **3D-(3,5/4)-trihydroxycyclohexane-1,2-dione hydrolase [EC:3.7.1.-]** | **0.002** | **0.932** |
| K03381 | catechol 1,2-dioxygenase [EC:1.13.11.1] | 0.037 | 0.746 |
| **K03782** | **catalase/peroxidase [EC:1.11.1.6 1.11.1.7]** | **0.013** | **0.859** |
| K03863 | vanillate monooxygenase [EC:1.14.13.82] | 0.037 | 0.746 |
| **K04118** | **pimeloyl-CoA dehydrogenase [EC:1.3.1.62]** | **0.019** | **0.812** |
| **K05358** | **quinate dehydrogenase (quinone) [EC:1.1.5.8];quinate dehydrogenase (pyrroloquinoline-quinone) [EC:1.1.99.25]** | **0.023** | **0.822** |
| **K06134** | **ubiquinone biosynthesis monooxygenase Coq7 [EC:1.14.13.-]** | **0.025** | **0.806** |
| **K06445** | **acyl-CoA dehydrogenase [EC:1.3.99.-]** | **0.012** | **0.853** |
| **K06447** | **succinylglutamic semialdehyde dehydrogenase [EC:1.2.1.71]** | **0.018** | **0.814** |
| **K07516** | **3-hydroxyacyl-CoA dehydrogenase [EC:1.1.1.35]** | **0.004** | **0.918** |
| K08685 | quinohemoprotein amine dehydrogenase [EC:1.4.98.1];amine dehydrogenase [EC:1.4.99.3] | 0.006 | 0.881 |
| **K09471** | **gamma-glutamylputrescine oxidase [EC:1.4.3.-]** | **0.013** | **0.855** |
| **K09472** | **gamma-glutamyl-gamma-aminobutyraldehyde dehydrogenase [EC:1.2.1.-]** | **0.005** | **0.902** |
| K10805 | acyl-CoA thioesterase II [EC:3.1.2.-] | 0.014 | 0.831 |
| **K10806** | **acyl-CoA thioesterase YciA [EC:3.1.2.-]** | **0.020** | **0.831** |
| K10946 | ammonia monooxygenase subunit C [EC:1.13.12.-] | 0.037 | 0.749 |
| K11755 | phosphoribosyl-ATP pyrophosphohydrolase / phosphoribosyl-AMP cyclohydrolase [EC:3.6.1.31 3.5.4.19] | 0.011 | 0.848 |
| K12251 | N-carbamoylputrescine amidase [EC:3.5.1.53] | 0.010 | 0.853 |
| **K12254** | **4-guanidinobutyraldehyde dehydrogenase / NAD-dependent aldehyde dehydrogenase [EC:1.2.1.54 1.2.1.-]** | **0.008** | **0.875** |
| **K12256** | **putrescine aminotransferase [EC:2.6.1.-]** | **0.009** | **0.884** |
| **K12524** | **bifunctional aspartokinase/homoserine dehydrogenase 1 [EC:2.7.2.4 1.1.1.3];bifunctional aspartokinase / homoserine dehydrogenase 1 [EC:2.7.2.4 1.1.1.3]** | **0.014** | **0.853** |
| K12979 | beta-hydroxylase [EC:1.14.11.-] | 0.026 | 0.783 |
| K13020 | UDP-D-GlcNAcA oxidase [EC:1.1.1.-] | 0.015 | 0.827 |
| **K13378** | **NADH dehydrogenase I subunit C/D [EC:1.6.5.3];NADH-quinone oxidoreductase subunit C/D [EC:1.6.5.3]** | **0.019** | **0.829** |
| **K13821** | **proline dehydrogenase / delta 1-pyrroline-5-carboxylate dehydrogenase [EC:1.5.99.8 1.5.1.12]** | **0.010** | **0.873** |
| **K13953** | **alcohol dehydrogenase, propanol-preferring [EC:1.1.1.1]** | **0.010** | **0.873** |
| **K13954** | **alcohol dehydrogenase [EC:1.1.1.1]** | **0.010** | **0.872** |
| **K14260** | **alanine-synthesizing transaminase [EC:2.6.1.66 2.6.1.2]** | **0.020** | **0.814** |
| K14267 | N-succinyldiaminopimelate aminotransferase [EC:2.6.1.17] | 0.044 | 0.729 |
| K14268 | 5-aminovalerate aminotransferase [EC:2.6.1.48] | 0.003 | 0.932 |
| K14519 | NADP-dependent aldehyde dehydrogenase [EC:1.2.1.4] | 0.003 | 0.915 |
| **Carbamazepine** | | | |
| **KEGG orthology number** | **Description** | **p value** | **Spearman’s ρ** |
| **K00005** | **glycerol dehydrogenase [EC:1.1.1.6]** | **0.049** | **0.775** |
| K00010 | myo-inositol 2-dehydrogenase [EC:1.1.1.18] | 0.037 | 0.850 |
| K00014 | shikimate dehydrogenase [EC:1.1.1.25];shikimate 5-dehydrogenase [EC:1.1.1.25] | 0.032 | 0.836 |
| K00018 | glycerate dehydrogenase [EC:1.1.1.29] | 0.043 | 0.796 |
| K00019 | 3-hydroxybutyrate dehydrogenase [EC:1.1.1.30] | 0.032 | 0.836 |
| **K00020** | **3-hydroxyisobutyrate dehydrogenase [EC:1.1.1.31]** | **0.042** | **0.816** |
| **K00024** | **malate dehydrogenase [EC:1.1.1.37]** | **0.036** | **0.832** |
| **K00029** | **malate dehydrogenase (oxaloacetate-decarboxylating)(NADP+) [EC:1.1.1.40]** | **0.033** | **0.848** |
| **K00031** | **isocitrate dehydrogenase [EC:1.1.1.42]** | **0.040** | **0.821** |
| K00055 | aryl-alcohol dehydrogenase [EC:1.1.1.90] | 0.046 | 0.786 |
| K00057 | glycerol-3-phosphate dehydrogenase (NAD(P)+) [EC:1.1.1.94] | 0.032 | 0.858 |
| **K00058** | **D-3-phosphoglycerate dehydrogenase [EC:1.1.1.95]** | **0.032** | **0.836** |
| **K00088** | **IMP dehydrogenase [EC:1.1.1.205]** | **0.038** | **0.818** |
| K00102 | D-lactate dehydrogenase (cytochrome) [EC:1.1.2.4] | 0.043 | 0.796 |
| **K00111** | **glycerol-3-phosphate dehydrogenase [EC:1.1.5.3]** | **0.041** | **0.803** |
| **K00116** | **malate dehydrogenase (quinone) [EC:1.1.5.4]** | **0.047** | **0.782** |
| **K00121** | **S-(hydroxymethyl)glutathione dehydrogenase / alcohol dehydrogenase [EC:1.1.1.284 1.1.1.1]** | **0.041** | **0.809** |
| **K00123** | **formate dehydrogenase, alpha subunit [EC:1.2.1.2]** | **0.038** | **0.825** |
| K00124 | formate dehydrogenase, beta subunit [EC:1.2.1.2] | 0.049 | 0.775 |
| **K00127** | **formate dehydrogenase, gamma subunit [EC:1.2.1.2]** | **0.044** | **0.796** |
| **K00128** | **aldehyde dehydrogenase (NAD+) [EC:1.2.1.3]** | **0.032** | **0.857** |
| K00130 | betaine-aldehyde dehydrogenase [EC:1.2.1.8] | 0.049 | 0.775 |
| **K00133** | **aspartate-semialdehyde dehydrogenase [EC:1.2.1.11]** | **0.040** | **0.811** |
| **K00134** | **glyceraldehyde 3-phosphate dehydrogenase [EC:1.2.1.12]** | **0.035** | **0.837** |
| **K00135** | **succinate-semialdehyde dehydrogenase (NADP+) [EC:1.2.1.16]** | **0.032** | **0.836** |
| **K00140** | **malonate-semialdehyde dehydrogenase (acetylating) / methylmalonate-semialdehyde dehydrogenase [EC:1.2.1.18 1.2.1.27];methylmalonate-semialdehyde dehydrogenase [EC:1.2.1.27]** | **0.038** | **0.820** |
| K00146 | phenylacetaldehyde dehydrogenase [EC:1.2.1.39] | 0.032 | 0.856 |
| **K00147** | **glutamate-5-semialdehyde dehydrogenase [EC:1.2.1.41]** | **0.039** | **0.814** |
| K00161 | pyruvate dehydrogenase E1 component subunit alpha [EC:1.2.4.1] | 0.043 | 0.796 |
| **K00163** | **pyruvate dehydrogenase E1 component [EC:1.2.4.1]** | **0.040** | **0.818** |
| **K00164** | **2-oxoglutarate dehydrogenase E1 component [EC:1.2.4.2]** | **0.038** | **0.822** |
| **K00166** | **2-oxoisovalerate dehydrogenase E1 component, alpha subunit [EC:1.2.4.4]** | **0.038** | **0.826** |
| K00228 | coproporphyrinogen III oxidase [EC:1.3.3.3] | 0.032 | 0.836 |
| **K00239** | **succinate dehydrogenase flavoprotein subunit [EC:1.3.99.1]** | **0.035** | **0.834** |
| **K00240** | **succinate dehydrogenase iron-sulfur protein [EC:1.3.99.1];succinate dehydrogenase iron-sulfur subunit [EC:1.3.99.1]** | **0.035** | **0.834** |
| **K00241** | **succinate dehydrogenase cytochrome b556 subunit;succinate dehydrogenase cytochrome b-556 subunit [EC:1.3.99.1];succinate dehydrogenase cytochrome b-556 subunit** | **0.040** | **0.814** |
| K00249 | acyl-CoA dehydrogenase [EC:1.3.99.3] | 0.032 | 0.858 |
| **K00252** | **glutaryl-CoA dehydrogenase [EC:1.3.99.7]** | **0.032** | **0.847** |
| K00253 | isovaleryl-CoA dehydrogenase [EC:1.3.99.10] | 0.032 | 0.856 |
| **K00259** | **alanine dehydrogenase [EC:1.4.1.1]** | **0.038** | **0.821** |
| **K00261** | **glutamate dehydrogenase (NAD(P)+) [EC:1.4.1.3]** | **0.035** | **0.832** |
| **K00262** | **glutamate dehydrogenase (NADP+) [EC:1.4.1.4]** | **0.040** | **0.820** |
| K00263 | leucine dehydrogenase [EC:1.4.1.9] | 0.032 | 0.856 |
| K00274 | monoamine oxidase [EC:1.4.3.4] | 0.049 | 0.775 |
| **K00281** | **glycine dehydrogenase [EC:1.4.4.2]** | **0.039** | **0.814** |
| **K00285** | **D-amino-acid dehydrogenase [EC:1.4.99.1]** | **0.044** | **0.797** |
| K00294 | 1-pyrroline-5-carboxylate dehydrogenase [EC:1.5.1.12] | 0.032 | 0.845 |
| **K00330** | **NADH-quinone oxidoreductase subunit A [EC:1.6.5.3];NADH dehydrogenase I subunit A [EC:1.6.5.3]** | **0.037** | **0.826** |
| K00331 | NADH dehydrogenase I subunit B [EC:1.6.5.3];NADH-quinone oxidoreductase subunit B [EC:1.6.5.3] | 0.032 | 0.836 |
| **K00332** | **NADH-quinone oxidoreductase subunit C [EC:1.6.5.3];NADH dehydrogenase I subunit C [EC:1.6.5.3]** | **0.032** | **0.847** |
| **K00333** | **NADH dehydrogenase I subunit D [EC:1.6.5.3];NADH-quinone oxidoreductase subunit D [EC:1.6.5.3]** | **0.037** | **0.837** |
| K00334 | NADH-quinone oxidoreductase subunit E [EC:1.6.5.3];NADH dehydrogenase I subunit E [EC:1.6.5.3] | 0.032 | 0.836 |
| **K00335** | **NADH dehydrogenase I subunit F [EC:1.6.5.3];NADH-quinone oxidoreductase subunit F [EC:1.6.5.3]** | **0.036** | **0.828** |
| **K00336** | **NADH-quinone oxidoreductase subunit G [EC:1.6.5.3];NADH dehydrogenase I subunit G [EC:1.6.5.3]** | **0.036** | **0.829** |
| **K00337** | **NADH dehydrogenase I subunit H [EC:1.6.5.3];NADH-quinone oxidoreductase subunit H [EC:1.6.5.3]** | **0.039** | **0.834** |
| **K00338** | **NADH-quinone oxidoreductase subunit I [EC:1.6.5.3];NADH dehydrogenase I subunit I [EC:1.6.5.3]** | **0.045** | **0.789** |
| **K00339** | **NADH dehydrogenase I subunit J [EC:1.6.5.3];NADH-quinone oxidoreductase subunit J [EC:1.6.5.3]** | **0.038** | **0.837** |
| **K00341** | **NADH dehydrogenase I subunit L [EC:1.6.5.3];NADH-quinone oxidoreductase subunit L [EC:1.6.5.3]** | **0.041** | **0.808** |
| **K00342** | **NADH-quinone oxidoreductase subunit M [EC:1.6.5.3];NADH dehydrogenase I subunit M [EC:1.6.5.3]** | **0.037** | **0.841** |
| **K00343** | **NADH dehydrogenase I subunit N [EC:1.6.5.3];NADH-quinone oxidoreductase subunit N [EC:1.6.5.3]** | **0.037** | **0.837** |
| **K00382** | **dihydrolipoamide dehydrogenase [EC:1.8.1.4]** | **0.038** | **0.821** |
| **K00404** | **cb-type cytochrome c oxidase subunit I [EC:1.9.3.1];cytochrome c oxidase cb-type subunit I [EC:1.9.3.1]** | **0.036** | **0.834** |
| **K00405** | **cb-type cytochrome c oxidase subunit II [EC:1.9.3.1];cytochrome c oxidase cb-type subunit II** | **0.036** | **0.830** |
| **K00406** | **cb-type cytochrome c oxidase subunit III [EC:1.9.3.1];cytochrome c oxidase cb-type subunit III** | **0.039** | **0.816** |
| **K00425** | **cytochrome d ubiquinol oxidase subunit I [EC:1.10.3.-];cytochrome bd-I oxidase subunit I [EC:1.10.3.-]** | **0.035** | **0.827** |
| **K00426** | **cytochrome d ubiquinol oxidase subunit II [EC:1.10.3.-];cytochrome bd-I oxidase subunit II [EC:1.10.3.-]** | **0.035** | **0.832** |
| K00432 | glutathione peroxidase [EC:1.11.1.9] | 0.032 | 0.845 |
| **K00457** | **4-hydroxyphenylpyruvate dioxygenase [EC:1.13.11.27]** | **0.035** | **0.833** |
| **K00459** | **nitronate monooxygenase [EC:1.13.12.16]** | **0.038** | **0.816** |
| K00466 | tryptophan 2-monooxygenase [EC:1.13.12.3] | 0.032 | 0.836 |
| K00499 | choline monooxygenase [EC:1.14.15.7] | 0.049 | 0.775 |
| **K00500** | **phenylalanine-4-hydroxylase [EC:1.14.16.1]** | **0.032** | **0.840** |
| **K00602** | **phosphoribosylaminoimidazolecarboxamide formyltransferase / IMP cyclohydrolase [EC:2.1.2.3 3.5.4.10]** | **0.036** | **0.832** |
| **K00627** | **pyruvate dehydrogenase E2 component (dihydrolipoamide acetyltransferase) [EC:2.3.1.12]** | **0.040** | **0.816** |
| **K00658** | **2-oxoglutarate dehydrogenase E2 component (dihydrolipoamide succinyltransferase) [EC:2.3.1.61]** | **0.039** | **0.817** |
| **K00817** | **histidinol-phosphate aminotransferase [EC:2.6.1.9]** | **0.032** | **0.836** |
| **K00821** | **acetylornithine/N-succinyldiaminopimelate aminotransferase [EC:2.6.1.11 2.6.1.17]** | **0.032** | **0.846** |
| **K00822** | **beta-alanine--pyruvate transaminase [EC:2.6.1.18]** | **0.038** | **0.816** |
| **K00823** | **4-aminobutyrate aminotransferase [EC:2.6.1.19]** | **0.038** | **0.817** |
| **K00826** | **branched-chain amino acid aminotransferase [EC:2.6.1.42]** | **0.041** | **0.806** |
| **K00831** | **phosphoserine aminotransferase [EC:2.6.1.52]** | **0.016** | **0.928** |
| K00836 | diaminobutyrate-2-oxoglutarate transaminase [EC:2.6.1.76] | 0.038 | 0.825 |
| **K01067** | **acetyl-CoA hydrolase [EC:3.1.2.1]** | **0.046** | **0.786** |
| **K01139** | **guanosine-3',5'-bis(diphosphate) 3'-pyrophosphohydrolase [EC:3.1.7.2]** | **0.032** | **0.836** |
| **K01442** | **choloylglycine hydrolase [EC:3.5.1.24]** | **0.046** | **0.786** |
| K01491 | methylenetetrahydrofolate dehydrogenase (NADP+) / methenyltetrahydrofolate cyclohydrolase [EC:1.5.1.5 3.5.4.9] | 0.032 | 0.836 |
| K01782 | 3-hydroxyacyl-CoA dehydrogenase / enoyl-CoA hydratase / 3-hydroxybutyryl-CoA epimerase [EC:1.1.1.35 4.2.1.17 5.1.2.3] | 0.032 | 0.836 |
| K01825 | 3-hydroxyacyl-CoA dehydrogenase / enoyl-CoA hydratase / 3-hydroxybutyryl-CoA epimerase / enoyl-CoA isomerase [EC:1.1.1.35 4.2.1.17 5.1.2.3 5.3.3.8] | 0.044 | 0.796 |
| K02274 | cytochrome c oxidase subunit I [EC:1.9.3.1] | 0.032 | 0.836 |
| K02275 | cytochrome c oxidase subunit II [EC:1.9.3.1] | 0.032 | 0.858 |
| K02297 | cytochrome o ubiquinol oxidase subunit II [EC:1.10.3.-] | 0.043 | 0.796 |
| **K02298** | **cytochrome o ubiquinol oxidase subunit I [EC:1.10.3.-]** | **0.039** | **0.811** |
| **K02299** | **cytochrome o ubiquinol oxidase subunit III [EC:1.10.3.-]** | **0.043** | **0.796** |
| **K02300** | **cytochrome o ubiquinol oxidase operon protein cyoD** | **0.038** | **0.816** |
| K02472 | UDP-N-acetyl-D-mannosaminuronic acid dehydrogenase [EC:1.1.1.-] | 0.032 | 0.858 |
| K02474 | UDP-N-acetyl-D-galactosamine dehydrogenase [EC:1.1.1.-] | 0.043 | 0.796 |
| **K02495** | **oxygen-independent coproporphyrinogen III oxidase [EC:1.3.99.22]** | **0.035** | **0.845** |
| **K03336** | **3D-(3,5/4)-trihydroxycyclohexane-1,2-dione hydrolase [EC:3.7.1.-]** | **0.038** | **0.826** |
| **K03782** | **catalase/peroxidase [EC:1.11.1.6 1.11.1.7]** | **0.039** | **0.820** |
| **K04118** | **pimeloyl-CoA dehydrogenase [EC:1.3.1.62]** | **0.032** | **0.889** |
| K05358 | quinate dehydrogenase (quinone) [EC:1.1.5.8];quinate dehydrogenase (pyrroloquinoline-quinone) [EC:1.1.99.25] | 0.032 | 0.836 |
| **K06134** | **ubiquinone biosynthesis monooxygenase Coq7 [EC:1.14.13.-]** | **0.032** | **0.858** |
| **K06445** | **acyl-CoA dehydrogenase [EC:1.3.99.-]** | **0.032** | **0.872** |
| **K07516** | **3-hydroxyacyl-CoA dehydrogenase [EC:1.1.1.35]** | **0.042** | **0.804** |
| K08685 | quinohemoprotein amine dehydrogenase [EC:1.4.98.1];amine dehydrogenase [EC:1.4.99.3] | 0.032 | 0.836 |
| **K09471** | **gamma-glutamylputrescine oxidase [EC:1.4.3.-]** | **0.038** | **0.827** |
| **K09472** | **gamma-glutamyl-gamma-aminobutyraldehyde dehydrogenase [EC:1.2.1.-]** | **0.032** | **0.847** |
| K10946 | ammonia monooxygenase subunit C [EC:1.13.12.-] | 0.032 | 0.900 |
| K12251 | N-carbamoylputrescine amidase [EC:3.5.1.53] | 0.043 | 0.796 |
| **K12254** | **4-guanidinobutyraldehyde dehydrogenase / NAD-dependent aldehyde dehydrogenase [EC:1.2.1.54 1.2.1.-]** | **0.036** | **0.826** |
| **K12256** | **putrescine aminotransferase [EC:2.6.1.-]** | **0.034** | **0.833** |
| K12524 | bifunctional aspartokinase/homoserine dehydrogenase 1 [EC:2.7.2.4 1.1.1.3];bifunctional aspartokinase / homoserine dehydrogenase 1 [EC:2.7.2.4 1.1.1.3] | 0.043 | 0.796 |
| K12979 | beta-hydroxylase [EC:1.14.11.-] | 0.032 | 0.858 |
| K13020 | UDP-D-GlcNAcA oxidase [EC:1.1.1.-] | 0.032 | 0.858 |
| K13378 | NADH dehydrogenase I subunit C/D [EC:1.6.5.3];NADH-quinone oxidoreductase subunit C/D [EC:1.6.5.3] | 0.038 | 0.816 |
| **K13821** | **proline dehydrogenase / delta 1-pyrroline-5-carboxylate dehydrogenase [EC:1.5.99.8 1.5.1.12]** | **0.037** | **0.827** |
| **K13953** | **alcohol dehydrogenase, propanol-preferring [EC:1.1.1.1]** | **0.040** | **0.811** |
| **K13954** | **alcohol dehydrogenase [EC:1.1.1.1]** | **0.036** | **0.828** |
| K14268 | 5-aminovalerate aminotransferase [EC:2.6.1.48] | 0.049 | 0.775 |
| K14519 | NADP-dependent aldehyde dehydrogenase [EC:1.2.1.4] | 0.032 | 0.836 |
| **Erythromycin** | | | |
| **KEGG orthology number** | **Description** | **p value** | **Spearman’s ρ** |
| K00010 | myo-inositol 2-dehydrogenase [EC:1.1.1.18] | 0.038 | -0.895 |
| K00012 | UDPglucose 6-dehydrogenase [EC:1.1.1.22] | 0.035 | 0.898 |
| K00013 | histidinol dehydrogenase [EC:1.1.1.23] | 0.043 | 0.881 |
| K00027 | malate dehydrogenase (oxaloacetate-decarboxylating) [EC:1.1.1.38] | 0.035 | 0.898 |
| **K00029** | **malate dehydrogenase (oxaloacetate-decarboxylating)(NADP+) [EC:1.1.1.40]** | **0.035** | **-0.910** |
| **K00031** | **isocitrate dehydrogenase [EC:1.1.1.42]** | **0.039** | **0.898** |
| **K00074** | **3-hydroxybutyryl-CoA dehydrogenase [EC:1.1.1.157]** | **0.036** | **0.897** |
| K00111 | glycerol-3-phosphate dehydrogenase [EC:1.1.5.3] | 0.035 | 0.898 |
| K00128 | aldehyde dehydrogenase (NAD+) [EC:1.2.1.3] | 0.043 | 0.881 |
| K00130 | betaine-aldehyde dehydrogenase [EC:1.2.1.8] | 0.033 | 0.921 |
| K00134 | glyceraldehyde 3-phosphate dehydrogenase [EC:1.2.1.12] | 0.035 | 0.915 |
| K00135 | succinate-semialdehyde dehydrogenase (NADP+) [EC:1.2.1.16] | 0.035 | -0.917 |
| **K00140** | **malonate-semialdehyde dehydrogenase (acetylating) / methylmalonate-semialdehyde dehydrogenase [EC:1.2.1.18 1.2.1.27];methylmalonate-semialdehyde dehydrogenase [EC:1.2.1.27]** | **0.035** | **0.898** |
| **K00161** | **pyruvate dehydrogenase E1 component subunit alpha [EC:1.2.4.1]** | **0.031** | **0.932** |
| K00162 | pyruvate dehydrogenase E1 component subunit beta [EC:1.2.4.1] | 0.043 | 0.881 |
| **K00164** | **2-oxoglutarate dehydrogenase E1 component [EC:1.2.4.2]** | **0.036** | **0.321** |
| K00167 | 2-oxoisovalerate dehydrogenase E1 component, beta subunit [EC:1.2.4.4] | 0.031 | -0.946 |
| **K00239** | **succinate dehydrogenase flavoprotein subunit [EC:1.3.99.1]** | **0.036** | **0.316** |
| K00241 | succinate dehydrogenase cytochrome b556 subunit;succinate dehydrogenase cytochrome b-556 subunit [EC:1.3.99.1];succinate dehydrogenase cytochrome b-556 subunit | 0.043 | 0.881 |
| K00252 | glutaryl-CoA dehydrogenase [EC:1.3.99.7] | 0.043 | -0.887 |
| K00253 | isovaleryl-CoA dehydrogenase [EC:1.3.99.10] | 0.031 | 0.940 |
| K00260 | glutamate dehydrogenase [EC:1.4.1.2] | 0.035 | 0.915 |
| K00261 | glutamate dehydrogenase (NAD(P)+) [EC:1.4.1.3] | 0.035 | 0.898 |
| K00294 | 1-pyrroline-5-carboxylate dehydrogenase [EC:1.5.1.12] | 0.035 | -0.912 |
| K00330 | NADH-quinone oxidoreductase subunit A [EC:1.6.5.3];NADH dehydrogenase I subunit A [EC:1.6.5.3] | 0.031 | -0.967 |
| K00334 | NADH-quinone oxidoreductase subunit E [EC:1.6.5.3];NADH dehydrogenase I subunit E [EC:1.6.5.3] | 0.031 | 0.932 |
| K00335 | NADH dehydrogenase I subunit F [EC:1.6.5.3];NADH-quinone oxidoreductase subunit F [EC:1.6.5.3] | 0.035 | 0.898 |
| K00336 | NADH-quinone oxidoreductase subunit G [EC:1.6.5.3];NADH dehydrogenase I subunit G [EC:1.6.5.3] | 0.043 | -0.883 |
| K00341 | NADH dehydrogenase I subunit L [EC:1.6.5.3];NADH-quinone oxidoreductase subunit L [EC:1.6.5.3] | 0.043 | -0.883 |
| **K00382** | **dihydrolipoamide dehydrogenase [EC:1.8.1.4]** | **0.035** | **0.898** |
| K00406 | cb-type cytochrome c oxidase subunit III [EC:1.9.3.1];cytochrome c oxidase cb-type subunit III | 0.031 | 0.932 |
| K00407 | cytochrome c oxidase cb-type subunit IV;cb-type cytochrome c oxidase subunit IV [EC:1.9.3.1] | 0.031 | 0.932 |
| K00457 | 4-hydroxyphenylpyruvate dioxygenase [EC:1.13.11.27] | 0.031 | 0.949 |
| K00459 | nitronate monooxygenase [EC:1.13.12.16] | 0.035 | -0.915 |
| K00602 | phosphoribosylaminoimidazolecarboxamide formyltransferase / IMP cyclohydrolase [EC:2.1.2.3 3.5.4.10] | 0.043 | 0.881 |
| **K00627** | **pyruvate dehydrogenase E2 component (dihydrolipoamide acetyltransferase) [EC:2.3.1.12]** | **0.036** | **0.903** |
| K00818 | acetylornithine aminotransferase [EC:2.6.1.11] | 0.031 | 0.949 |
| K00819 | ornithine--oxo-acid transaminase [EC:2.6.1.13] | 0.043 | 0.881 |
| K00820 | glucosamine--fructose-6-phosphate aminotransferase (isomerizing) [EC:2.6.1.16] | 0.035 | -0.915 |
| K00821 | acetylornithine/N-succinyldiaminopimelate aminotransferase [EC:2.6.1.11 2.6.1.17] | 0.035 | 0.915 |
| **K00823** | **4-aminobutyrate aminotransferase [EC:2.6.1.19]** | **0.035** | **0.907** |
| K00832 | aromatic-amino-acid transaminase [EC:2.6.1.57] | 0.045 | 0.879 |
| K00840 | succinylornithine aminotransferase [EC:2.6.1.81] | 0.035 | 0.898 |
| K01066 | esterase / lipase [EC:3.1.1.-] | 0.043 | 0.881 |
| K01451 | hippurate hydrolase [EC:3.5.1.32] | 0.043 | 0.881 |
| K01491 | methylenetetrahydrofolate dehydrogenase (NADP+) / methenyltetrahydrofolate cyclohydrolase [EC:1.5.1.5 3.5.4.9] | 0.035 | 0.898 |
| **K01825** | **3-hydroxyacyl-CoA dehydrogenase / enoyl-CoA hydratase / 3-hydroxybutyryl-CoA epimerase / enoyl-CoA isomerase [EC:1.1.1.35 4.2.1.17 5.1.2.3 5.3.3.8]** | **0.035** | **0.898** |
| K02277 | cytochrome c oxidase subunit IV [EC:1.9.3.1] | 0.035 | 0.898 |
| K02495 | oxygen-independent coproporphyrinogen III oxidase [EC:1.3.99.22] | 0.031 | -0.933 |
| K04072 | acetaldehyde dehydrogenase / alcohol dehydrogenase [EC:1.2.1.10 1.1.1.1] | 0.043 | 0.881 |
| K05599 | anthranilate 1,2-dioxygenase (deaminating, decarboxylating) large subunit [EC:1.14.12.1] | 0.035 | 0.912 |
| K06193 | phosphonoacetate hydrolase [EC:3.11.1.2] | 0.043 | 0.881 |
| K07127 | 5-hydroxyisourate hydrolase [EC:3.5.2.17] | 0.043 | 0.881 |
| K09699 | 2-oxoisovalerate dehydrogenase E2 component (dihydrolipoyl transacylase) [EC:2.3.1.168] | 0.045 | -0.879 |
| K12256 | putrescine aminotransferase [EC:2.6.1.-] | 0.035 | 0.898 |
| K12979 | beta-hydroxylase [EC:1.14.11.-] | 0.031 | -0.950 |
| K13821 | proline dehydrogenase / delta 1-pyrroline-5-carboxylate dehydrogenase [EC:1.5.99.8 1.5.1.12] | 0.035 | -0.900 |
| **K13953** | **alcohol dehydrogenase, propanol-preferring [EC:1.1.1.1]** | **0.035** | **0.898** |
| K14260 | alanine-synthesizing transaminase [EC:2.6.1.66 2.6.1.2] | 0.043 | 0.881 |

## 2.5 Gene set enrichment analysis

## Using the *P*-values from these associations in Table S6, we performed a gene set enrichment analysis. In the gene set enrichment analysis, categories of functions, rather than individual genes, were tested for associations with pharmaceutical biotransformation. We excluded erythromycin from the gene set enrichment analysis because it did not associate with dilution, we would expect a different set of functions to associate with erythromycin transformation. The significantly associated functions and their associated pathways that emerged from the gene set enrichment analysis are shown in Table S7 and in Figure S6.

**
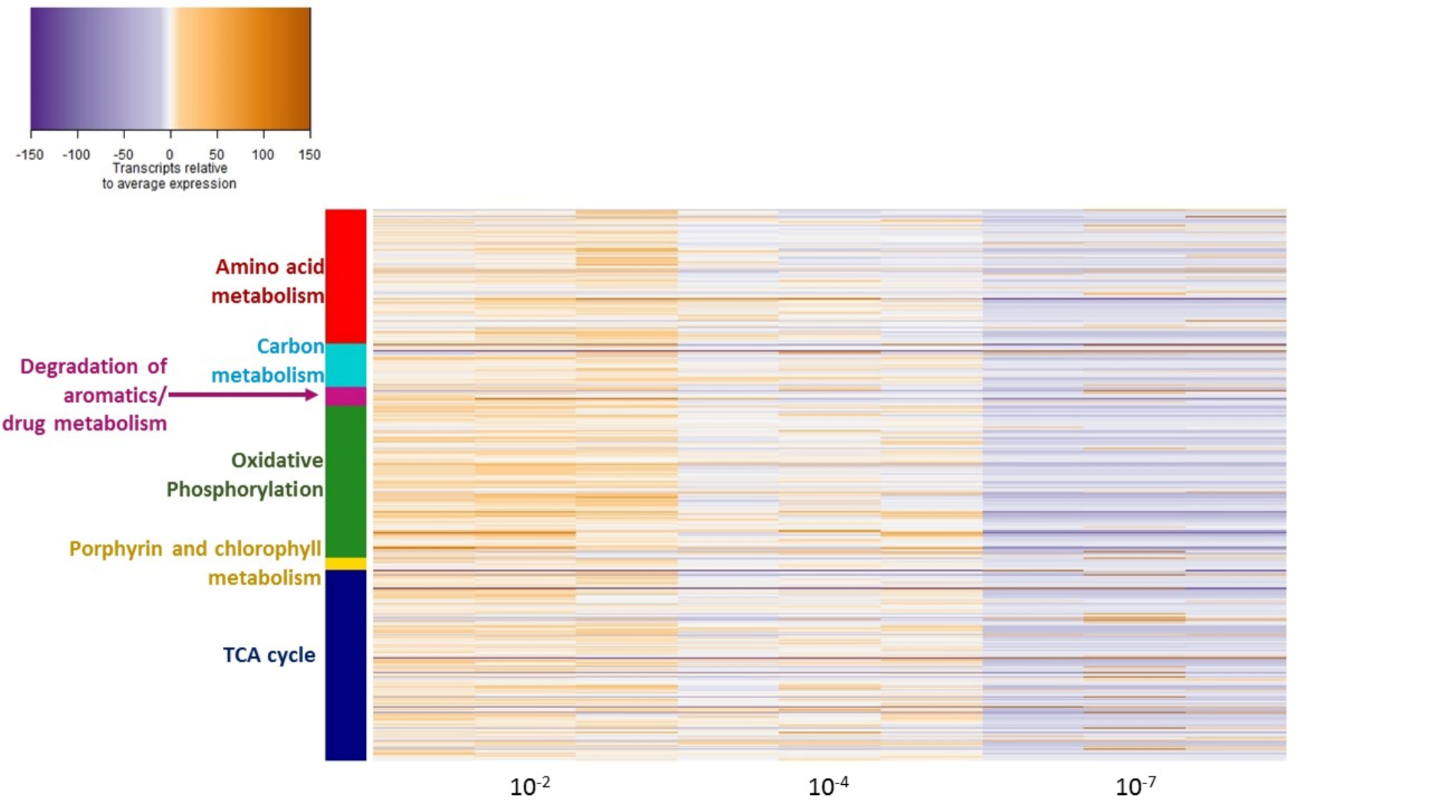
**

**Figure S6**. Relative expression of significantly differentially expressed genes (likelihood ratio, Benjamini-Hochberg adjusted *P*<0.05) that were annotated with KO terms associated with pharmaceutical transformation based on the gene enrichment analysis.

##

**Table S7.** KEGG identifiers for functions that associated with transformation across all pharmaceutical compounds based on gene enrichment analysis.

| **KEGG Orthology** | **Description** | **Pathway** | **Citations**  (Culture type, contaminant) |
| --- | --- | --- | --- |
| K00020 | 3-hydroxyisobutyrate dehydrogenase [EC:1.1.1.31] | amino acid metabolism | Pure Culture, crude oil (Nie *et al.*, 2012) |
| K00029 | malate dehydrogenase (oxaloacetate-decarboxylating)(NADP+) [EC:1.1.1.40] | amino acid metabolism | Pure culture, E2 (Li *et al.*, 2012) |
| K00133 | aspartate-semialdehyde dehydrogenase [EC:1.2.1.11] | amino acid metabolism | Pure culture, chloronitrobenzene (Zhang *et al.*, 2009) |
| K00135 | succinate-semialdehyde dehydrogenase (NADP+) [EC:1.2.1.16] | amino acid metabolism | Pure culture, chloronitrobenzene (Zhang *et al.*, 2009) |
| K00140 | malonate-semialdehyde dehydrogenase (acetylating) / methylmalonate-semialdehyde dehydrogenase [EC:1.2.1.18 1.2.1.27];methylmalonate-semialdehyde dehydrogenase [EC:1.2.1.27] | amino acid metabolism | Pure culture, chloronitrobenzene (Zhang *et al.*, 2009) |
| K00259 | alanine dehydrogenase [EC:1.4.1.1] | amino acid metabolism |  |
| K00281 | glycine dehydrogenase [EC:1.4.4.2] | amino acid metabolism |  |
| K00831 | phosphoserine aminotransferase [EC:2.6.1.52] | amino acid metabolism |  |
| K03782 | catalase/peroxidase [EC:1.11.1.6 1.11.1.7] | amino acid metabolism | Enzyme isolate, pharmaceuticals (Stadlmair *et al.*, 2017); Review paper (Azevedo *et al.*, 2003) |
| K12256 | putrescine aminotransferase [EC:2.6.1.-] | amino acid metabolism |  |
| K13821 | proline dehydrogenase / delta 1-pyrroline-5-carboxylate dehydrogenase [EC:1.5.99.8 1.5.1.12] | amino acid metabolism |  |
| K00123 | formate dehydrogenase, alpha subunit [EC:1.2.1.2] | carbon metabolism |  |
| K00134 | glyceraldehyde 3-phosphate dehydrogenase [EC:1.2.1.12] | carbon metabolism | Pure culture, dioxane (Grostern *et al.*, 2012) |
| K00252 | glutaryl-CoA dehydrogenase [EC:1.3.99.7] | carbon metabolism |  |
| K01825 | 3-hydroxyacyl-CoA dehydrogenase / enoyl-CoA hydratase / 3-hydroxybutyryl-CoA epimerase / enoyl-CoA isomerase [EC:1.1.1.35 4.2.1.17 5.1.2.3 5.3.3.8] | carbon metabolism |  |
| K00088 | IMP dehydrogenase [EC:1.1.1.205] | degradation of aromatic compounds/ drug metabolism |  |
| K13954 | alcohol dehydrogenase [EC:1.1.1.1] | degradation of aromatic compounds/ drug metabolism | Pure culture, dioxane (Gedalanga *et al.*, 2014) |
| K00335 | NADH dehydrogenase I subunit F [EC:1.6.5.3];NADH-quinone oxidoreductase subunit F [EC:1.6.5.3] | oxidative phosphorylation | Pure culture, dioxane (Grostern *et al.*, 2012) |
| K00336 | NADH-quinone oxidoreductase subunit G [EC:1.6.5.3];NADH dehydrogenase I subunit G [EC:1.6.5.3] | oxidative phosphorylation |  |
| K00337 | NADH dehydrogenase I subunit H [EC:1.6.5.3];NADH-quinone oxidoreductase subunit H [EC:1.6.5.3] | oxidative phosphorylation | Pure culture, dioxane (Grostern *et al.*, 2012) |
| K00341 | NADH dehydrogenase I subunit L [EC:1.6.5.3];NADH-quinone oxidoreductase subunit L [EC:1.6.5.3] | oxidative phosphorylation | Pure culture, dioxane (Grostern *et al.*, 2012) |
| K00343 | NADH dehydrogenase I subunit N [EC:1.6.5.3];NADH-quinone oxidoreductase subunit N [EC:1.6.5.3] | oxidative phosphorylation | Pure culture, dioxane (Grostern *et al.*, 2012) |
| K00404 | cb-type cytochrome c oxidase subunit I [EC:1.9.3.1];cytochrome c oxidase cb-type subunit I [EC:1.9.3.1] | oxidative phosphorylation | Surface water biofilm, pharmaceuticals (Yergeau *et al.*, 2012) |
| K00405 | cb-type cytochrome c oxidase subunit II [EC:1.9.3.1];cytochrome c oxidase cb-type subunit II | oxidative phosphorylation | Surface water biofilm, pharmaceuticals (Yergeau *et al.*, 2012) |
| K00406 | cb-type cytochrome c oxidase subunit III [EC:1.9.3.1];cytochrome c oxidase cb-type subunit III | oxidative phosphorylation | Surface water biofilm, pharmaceuticals (Yergeau *et al.*, 2012) |
| K00425 | cytochrome d ubiquinol oxidase subunit I [EC:1.10.3.-];cytochrome bd-I oxidase subunit I [EC:1.10.3.-] | oxidative phosphorylation |  |
| K00426 | cytochrome d ubiquinol oxidase subunit II [EC:1.10.3.-];cytochrome bd-I oxidase subunit II [EC:1.10.3.-] | oxidative phosphorylation |  |
| K02297 | cytochrome o ubiquinol oxidase subunit II [EC:1.10.3.-] | oxidative phosphorylation |  |
| K02298 | cytochrome o ubiquinol oxidase subunit I [EC:1.10.3.-] | oxidative phosphorylation |  |
| K02495 | oxygen-independent coproporphyrinogen III oxidase [EC:1.3.99.22] | Porphyrin and chlorophyll metabolism |  |
| K00024 | malate dehydrogenase [EC:1.1.1.37] | TCA cycle | Pure culture, E2 (Li *et al.*, 2012) |
| K00031 | isocitrate dehydrogenase [EC:1.1.1.42] | TCA cycle | Pure culture, E2 (Li *et al.*, 2012) |
| K00116 | malate dehydrogenase (quinone) [EC:1.1.5.4] | TCA cycle | Pure culture, E2 (Li *et al.*, 2012) |
| K00164 | 2-oxoglutarate dehydrogenase E1 component [EC:1.2.4.2] | TCA cycle |  |
| K00239 | succinate dehydrogenase flavoprotein subunit [EC:1.3.99.1] | TCA cycle |  |
| K00240 | succinate dehydrogenase iron-sulfur protein [EC:1.3.99.1];succinate dehydrogenase iron-sulfur subunit [EC:1.3.99.1] | TCA cycle |  |
| K00241 | succinate dehydrogenase cytochrome b556 subunit;succinate dehydrogenase cytochrome b-556 subunit [EC:1.3.99.1];succinate dehydrogenase cytochrome b-556 subunit | TCA cycle |  |
| K00382 | dihydrolipoamide dehydrogenase [EC:1.8.1.4] | TCA cycle | Pure culture, dioxane (Grostern *et al.*, 2012) |
| K00658 | 2-oxoglutarate dehydrogenase E2 component (dihydrolipoamide succinyltransferase) [EC:2.3.1.61] | TCA cycle |  |

## References

Anders, S., Pyl, P.T., and Huber, W. (2014) HTSeq A Python framework to work with high-throughput sequencing data. *Bioinformatics* **31**: 166–169.

Azevedo, A.M., Martins, V.C., Prazeres, D.M.F., Vojinović, V., Cabral, J.M.S., and Fonseca, L.P. (2003) Horseradish peroxidase: A valuable tool in biotechnology. *Biotechnol. Annu. Rev.* **9**: 199–247.

Benjamini, Y. and Hochberg, Y. (1995) Controlling the false discovery rate: a practical and powerful approach to multiple testing. *J. R. Stat. Soc. Ser. B* 289–300.

Cole, J.R., Wang, Q., Cardenas, E., Fish, J., Chai, B., Farris, R.J., et al. (2009) The Ribosomal Database Project: improved alignments and new tools for rRNA analysis. *Nucleic Acids Res.* **37**: D141–D145.

EAWAG (2016) Biocatalysis/Biodegradation Database.

Ellis, L.B.M., Roe, D., and Wackett, L.P. (2006) The University of Minnesota biocatalysis/biodegradation database: the first decade. *Nucleic Acids Res.* **34**: D517–D521.

Fayad, P.B., Prévost, M., and Sauvé, S. (2013) On-line solid-phase extraction coupled to liquid chromatography tandem mass spectrometry optimized for the analysis of steroid hormones in urban wastewaters. *Talanta* **115**: 349–360.

Gedalanga, P.B., Pornwongthong, P., Mora, R., Chiang, S.Y.D., Baldwin, B., Ogles, D., and Mahendraa, S. (2014) Identification of biomarker genes to predict biodegradation of 1,4-dioxane. *Appl. Environ. Microbiol.* **80**: 3209–3218.

Grady, C.P.L., Daigger, G.T., Love, N.G., and Filipe, C.D.M. (2011) Biological Wastewater Treatment 3rd ed. CRC Press, Boca Raton.

Grostern, A., Sales, C.M., Zhuang, W.Q., Erbilgin, O., and Alvarez-Cohena, L. (2012) Glyoxylate metabolism is a key feature of the metabolic degradation of 1,4-dioxane by Pseudonocardia dioxanivorans strain CB1190. *Appl. Environ. Microbiol.* **78**: 3298–3308.

Huang, M., Li, Y., and Gu, G. (2010) Chemical composition of organic matters in domestic wastewater. *Desalination* **262**: 36–42.

Joshi, N.A. and Fass, J.N. (2011) Sickle: A sliding-window, adaptive, quality-based trimming tool for FastQ files (Version 1.33)[Software].

Kozich, J.J., Westcott, S.L., Baxter, N.T., Highlander, S.K., and Schloss, P.D. (2013) Development of a Dual-Index Sequencing Strategy and Curation Pipeline for Analyzing Amplicon Sequence Data on the MiSeq Illumina Sequencing Platform. *Appl. Environ. Microbiol.* **79**: 5112–5120.

Li, H. and Durbin, R. (2009) Fast and accurate short read alignment with Burrows-Wheeler transform. *Bioinformatics* **25**: 1754–60.

Li, H., Handsaker, B., Wysoker, A., Fennell, T., Ruan, J., Homer, N., et al. (2009) The Sequence Alignment/Map format and SAMtools. *Bioinformatics* **25**: 2078–2079.

Li, Z., Nandakumar, R., Madayiputhiya, N., and Li, X. (2012) Proteomic analysis of 17beta-estradiol degradation by Stenotrophomonas maltophilia. *Environ. Sci. Technol.* **46**: 5947–5955.

Love, M.I., Huber, W., and Anders, S. (2014) Moderated estimation of fold change and dispersion for RNA-seq data with DESeq2. *Genome Biol.* **15**: 550.

Nie, Y., Tang, Y.Q., Li, Y., Chi, C.Q., Cai, M., and Wu, X.L. (2012) The genome sequence of polymorphum gilvum SL003B-26A1 T reveals its genetic basis for crude oil degradation and adaptation to the saline soil. *PLoS One* **7**:.

Oksanen, J., Kindt, R., Legendre, P., O’Hara, B., Stevens, M.H.H., Oksanen, M.J., and Suggests, M. (2007) The vegan package. *Community Ecol. Packag.* 631–637.

Peng, Y., Leung, H.C.M., Yiu, S.M., and Chin, F.Y.L. (2012) IDBA-UD: a de novo assembler for single-cell and metagenomic sequencing data with highly uneven depth. *Bioinformatics* **28**: 1420–8.

Schloss, P.D., Westcott, S.L., Ryabin, T., Hall, J.R., Hartmann, M., Hollister, E.B., et al. (2009) Introducing mothur: Open-Source, Platform-Independent, Community-Supported Software for Describing and Comparing Microbial Communities. *Appl Env. Microbiol* **75**: 7537–7541.

Stadlmair, L.F., Letzel, T., Drewes, J.E., and Graßmann, J. (2017) Mass spectrometry based in vitro assay investigations on the transformation of pharmaceutical compounds by oxidative enzymes. *Chemosphere* **174**: 466–477.

Venables, W.N., Smith, D.M., and R Development Core Team (2004) An Introduction to R.

Webster, T.M., Smith, A.L., Reddy, R.R., Pinto, A.J., Hayes, K.F., and Raskin, L. (2016) Anaerobic microbial community response to methanogenic inhibitors 2-bromoethanesulfonate and propynoic acid. *Microbiologyopen* **5**: 537–550.

Yergeau, E., Sanschagrin, S., Waiser, M.J., Lawrence, J.R., and Greer, C.W. (2012) Sub-inhibitory concentrations of different pharmaceutical products affect the meta-transcriptome of river biofilm communities cultivated in rotating annular reactors. *Environ. Microbiol. Rep.* **4**: 350–359.

Yu, G., Wang, L.-G., Han, Y., and He, Q.-Y. (2012) clusterProfiler: an R Package for Comparing Biological Themes Among Gene Clusters. *Omi. A J. Integr. Biol.* **16**: 284–287.

Zavaleta, E.S., Pasari, J.R., Hulvey, K.B., and Tilman, G.D. (2010) Sustaining multiple ecosystem functions in grassland communities requires higher biodiversity. *Proc. Natl. Acad. Sci.* **107**: 1443–1446.

Zhang, Y., Wu, J.F., Zeyer, J., Meng, B., Liu, L., Jiang, C.Y., et al. (2009) Proteomic and molecular investigation on the physiological adaptation of Comamonas sp. strain CNB-1 growing on 4-chloronitrobenzene. *Biodegradation* **20**: 55–66.
